# Quantifying optical scattering losses in pump–probe serial femtosecond crystallography experiments

**DOI:** 10.1107/S2052252526005403

**Published:** 2026-07-16

**Authors:** Thomas R. M. Barends, Alexander Gorel, Stanisław Niziński, Martin V. Appleby, Marco Kloos, Soshichiro Nagano, Mario Hilpert, Florian Dworkowski, Claudio Cirelli, Emma V. Beale, Elisabeth Hartmann, Camila Bacellar, Robert L. Shoeman, R. Bruce Doak, John H. Beale, Philip J. M. Johnson, Ilme Schlichting

**Affiliations:** a Max Planck Institute for Medical Research Jahnstraße 29 Heidelberg 69120 Germany; b Swiss Light Source Paul Scherrer Institute Forschungsstraße 111 Villigen 5232 Switzerland; c European XFEL Holzkoppel 4 Schenefeld 22869 Germany; d SwissFEL Paul Scherrer Institute Forschungsstraße 111 Villigen 5232 Switzerland; Uppsala University, Sweden

**Keywords:** time-resolved serial crystallography, serial femtosecond crystallography, pump–probe studies, light scattering, light contamination, membrane protein crystals, fixed targets, high-viscosity jets, femtosecond studies, molecular movies, protein structure

## Abstract

Ultrafast pump–probe serial femtosecond crystallography (SFX) experiments are typically performed with extremely high excitation laser fluence to maximize the occupancy of the light-induced state. This has been justified by assuming that a dominant fraction (up to 99%) of the pump laser light is scattered by the jetting medium, strongly reducing the pump laser fluence in the crystals which, therefore, do not absorb multiple photons and thus undergo the biologically relevant single-photon photoreaction. However, this notion is strongly contested in the field. To address the issue of pump laser scattering losses in high viscosity jets, we performed time-resolved SFX experiments on fatty acid photo­decarboxyl­ase crystals using high-viscosity extrusion (HVE) and both patterned and non-patterned sheet-on-sheet (SOS) fixed-target sample delivery systems on the Cristallina-MX instrument at SwissFEL. Tightly focused pump laser beams were used to mitigate light contamination (the undesired accidental illumination of neighboring crystals) in SOS chips, paving the way for their use in pump–probe investigations of lipidic-cubic-phase-grown membrane protein crystals. Determination of the light-induced reaction intermediate occupancies enabled a comparison of the influence of the delivery method on the pump laser fluence reaching the crystals. We show that optically transparent high-viscosity jets and chip crystal delivery methods result in photoproduct yields that approach 70–80% and 60–70%, respectively, of computationally derived yields that take into account excitation probabilities. These results demonstrate that clear viscous jets scatter pump laser light far less than has been claimed. Consequently, the use of excessive photoexcitation energy densities is neither needed nor justified in optically pumped time-resolved SFX experiments when using appropriately sized crystals.

## Abstract 2

Similar yields of the optically excited fractions of fatty acid photodecarboxyl­ase crystals in pump–probe serial femtosecond crystallography (SFX) experiments using both fixed-target and high-viscosity extrusion (HVE) sample delivery approaches show that pump light scattering reduces the photon flux reaching the crystals in transparent high-viscosity jets by 20–40%, depending on delivery method. This experimental result contradicts previous reports of up to 99% scattering losses, which were argued to justify the use of extremely high pump laser energy densities without entering multiphoton absorption regimes.

## 1. Introduction

Time-resolved diffraction studies of light-sensitive molecules aim to provide structural and mechanistic insight into a (biochemical) reaction. This could be a naturally light-driven reaction in a photosensory protein, or a reaction made possible by rendering a system light sensitive through the addition of a photosensitive protecting (cage) group to a substrate or small-molecule activator. In all cases, the experimental scheme involves a photoactivating UV–Vis pump laser pulse followed by a temporally delayed X-ray probe pulse. In serial data acquisition schemes, the sample is rapidly replenished for the next pair of pump and probe pulses, often by means of a liquid or high-viscosity jet. A crucial component for the success of the experiment is a high occupancy of the light-induced reaction intermediate in the case of naturally light-sensitive reactions, and of the desired product in the case of uncaging reactions. This yield depends on many parameters, but the number of pump photons impinging on the chromophore is a crucial factor.

While this number is nominally straightforward to calculate, there is considerable disagreement in the community as to how much of the incident pump light reaches the crystals. Estimates of scattering losses range from ≤20% to 99% of the light failing to reach the crystals in the interaction region (Arnlund *et al.*, 2014 ▸; Nogly *et al.*, 2018 ▸; Nass Kovacs *et al.*, 2019 ▸; Claesson *et al.*, 2020 ▸; Grünbein *et al.*, 2020 ▸; Carrillo *et al.*, 2021 ▸). Knowing whether a significant fraction of the incident pump laser photons are really scattered is important, because strong scattering losses have been used to justify the very high excitation power densities applied in ultra-fast time-resolved serial femtosecond crystallography (TR-SFX) experiments and to argue that, despite these high densities, the photoreaction took place under single-photon excitation conditions (Nogly *et al.*, 2018 ▸; Claesson *et al.*, 2020 ▸; Carrillo *et al.*, 2021 ▸). Single-photon conditions are highly desirable, in particular for excitation with intense ultra-short pulses, as multiphoton absorption and/or nonlinear optical processes can result in significant deviations from the biologically relevant low-fluence photochemistry through excitation to higher electronic states (even leading to ionization). For longer excitation pulses, population cycling through sequential absorption (Miller *et al.*, 2020 ▸; Besaw & Miller, 2023 ▸; Heyne *et al.*, 2024 ▸; Do *et al.*, 2024 ▸; Barends *et al.*, 2024 ▸) or local sample heating can also occur. These processes can significantly modify the structure, dynamics and kinetics of the system under study. Indeed, even at a relatively modest excitation fluence of ∼20 mJ cm^−2^ used to photoinduce ligand release in carb­oxy­myoglobin microcrystals (Barends *et al.*, 2024 ▸), the observed characteristics and kinetics of ligand release and the amplitudes of molecular displacements differ significantly from those obtained when photoexciting in the single-photon absorption regime at 5 mJ cm^−2^. To determine whether the excessive excitation conditions typical for ultrafast TR-SFX experiments are really required to compensate for optical losses, it is imperative to know the true extent of optical losses of pump laser energy in TR-SFX experiments. The only way to quantify this is through experimental characterization. Such characterization would provide a crucial part of the data that, alongside other information such as extinction coefficients, crystal- and laser spot dimensions *etc.*, are required for the calculation of appropriate pump laser fluences so as to avoid multiphoton excitation and, ultimately, erroneous interpretations of the derived structural dynamics.

Unfortunately, it is not straightforward to directly measure either the optical losses caused by scattering, or the spot size of the pump beam within a liquid jet. However, the fractional occupancy of photoexcited molecules can be quantified by crystallography and used as an empirical measure of the effective pump laser energy density at the sample position. This effective pump laser energy density can differ from the incident energy density due to scattering and other losses that may depend on the sample delivery method.

The various sample delivery methods are highly diverse in their geometric and optical properties and, therefore, in how much they can affect the amount of light reaching the crystals. In jet-based delivery methods, crystals are fully immersed in a column of transporting liquid, resulting in considerable potential for scattering or even refocusing of the beam onto the crystals or some point outside the jet (Grünbein *et al.*, 2020 ▸). On the other hand, patterned fixed targets isolate crystals in wells, where they are surrounded by very little additional medium that could scatter or refocus an optical excitation beam (Mueller *et al.*, 2015 ▸). By contrast, sheet-on-sheet (SOS) chips (Doak *et al.*, 2018 ▸; Doak *et al.*, 2024 ▸) are non-patterned fixed targets where crystals are surrounded by their growth medium, either a liquid or lipidic cubic phase (LCP) in the case of many membrane protein crystals. Thus, a direct quantitative comparison of activation levels of optically excited crystals in jets *versus* fixed targets (both patterned and non-patterned SOS) should enable the characterization of the influence that the jetting medium and crystal delivery geometry have on pump light scattering.

To this end, we performed TR-SFX experiments using microcrystals of the photoenzyme fatty acid photo­decarboxyl­ase (FAP) that catalyzes the irreversible light-induced de­carboxyl­ation of fatty acids (Sorigue *et al.*, 2021 ▸). A structurally well defined intermediate forms with a time constant of ∼300 ps that decays on a microsecond timescale (Sorigue *et al.*, 2021 ▸). Determining the occupancies of the cleaved and uncleaved fatty acid substrate allows for a straightforward determination of the photoexcitation yield of the reaction intermediate and serves as a proxy for the fluence reaching the interaction region. Here, we show that the photoexcitation yields obtained with fixed targets and high-viscosity extrusion (HVE) are comparable and that neither the cylindrical geometry of jets nor the optically clear jetting medium [LCP or hy­droxy­ethyl cellulose (HEC)] results in pump light scattering and thus fluence reduction strong enough to reduce photoproduct yields even by just one order of magnitude, in line with recent all-optical investigations (Niziński *et al.*, 2025 ▸). Indeed, we show experimentally that the losses are around 20–30%, *i.e.* at the lower end of the range of estimates found in the literature. Our findings have implications for the interpretation of past experiments and provide guidance for the design of future TR-SFX pump–probe experiments.

## 2. Materials and methods

### 2.1. Sample preparation

FAP was purified (Sorigue *et al.*, 2021 ▸) and crystallized in batch using seeding approaches as described previously (Shoeman *et al.*, 2023 ▸) under safe yellow or red light. The longest dimension of the crystals was ∼20 ± 5 µm [Fig. S1(*a*) in the supporting information]. LCP for embedding FAP microcrystals was prepared by mixing 60 µl of monoolein (42°C) and 35–40 µl of a solution consisting of 76 m*M* Bis Tris Propane pH 7.5, 18%(*w*/*v*) pluronic F-108 and 35%(*w*/*v*) PEG 3350 using coupled Hamilton Gastight syringes until the mixture became transparent. The HEC matrix used for embedding the FAP microcrystals was prepared by mixing solid HEC powder (280 mg) and 2 ml of 50 m*M* Bis Tris Propane pH 7.5 and 25%(*w*/*v*) PEG 3350. Once the suspension started clearing (but was still granular) it was pipetted into 100 µl Hamilton Gastight syringes and left to swell for at least one day. The HEC concentration was nominally ∼14%(*w*/*v*).

The protein microcrystal suspensions were pelleted by gentle centrifugation before embedding in the HEC or LCP viscous matrix. A portion (12 µl) of the resulting rather dry crystalline pellet was mixed until homogeneous with 95–100 µl of the transparent viscous matrix material (LCP or HEC, see above) using coupled Hamilton Gastight syringes. For SFX data collection the contents of three such syringes were mixed until homogeneous, pooled and filled into a custom-built large-volume HVE injector (Doak *et al.*, in preparation).

FAP microcrystals suspended in artificial mother liquor [50 m*M* Bis Tris Propane pH 7.5, 25%(*w*/*v*) PEG 3350] were mounted on patterned opaque micro-structured polymer (MISP) chips (Carrillo *et al.*, 2023 ▸; Gotthard *et al.*, 2024 ▸) with hole sizes of ∼5–7 µm as described previously (Gotthard *et al.*, 2024 ▸). A 250 µl aliquot of crystalline slurry (8 × 10^5^ crystals ml^−1^) in artificial mother liquor was spread over the open wells surface of the chip. Excess solution was removed by briefly applying a vacuum to the flat reverse side. The chip was then sandwiched between two sheets of (loosely stretched) 6 µm Mylar, placed inside the holder and immediately taken to the endstation for data collection. To mount the crystals on SOS chips, 25–30 µl of the LCP/FAP microcrystal mixture was dabbed in discontinuous lines or droplets onto the lower membrane of the SOS chip before adding the upper membrane (both 6 µm thick Mylar foils). A homogeneous sample layer was made using the SOS sample press as described previously (Doak *et al.*, 2024 ▸). The Mylar foils were stretched taut and the crystal-containing LCP layer was 25 ± 5 µm thick.

### 2.2. Crystal delivery, photoexcitation, SFX data collection and analysis

#### 2.2.1. Crystal delivery

**Figure 1:**
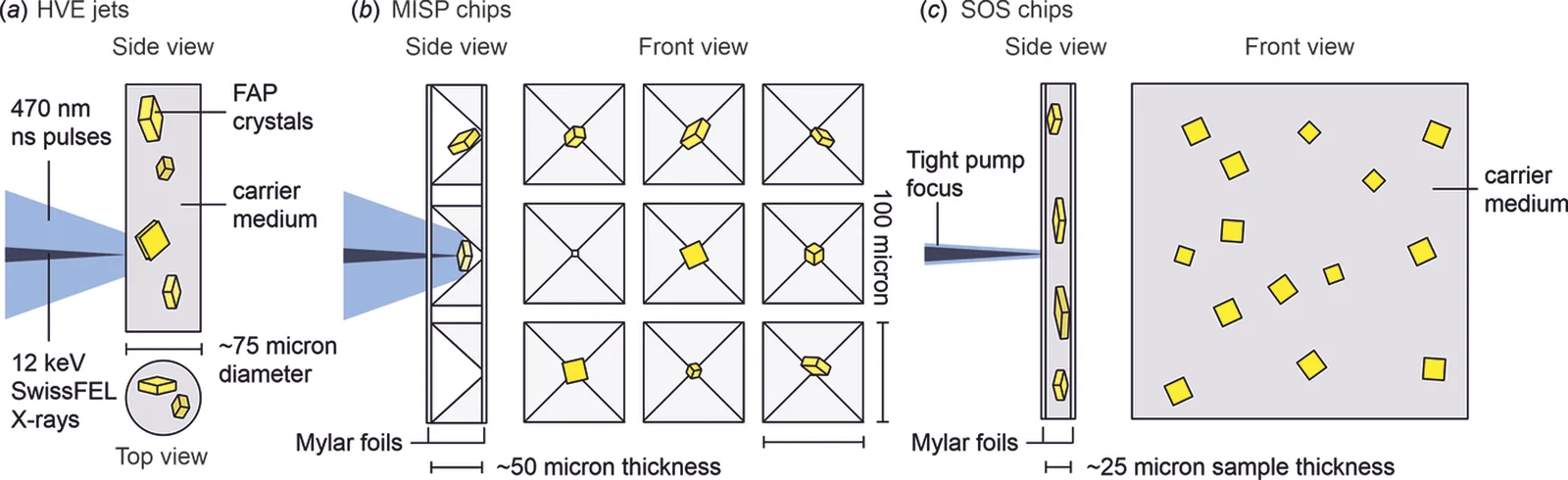
The sample delivery systems used in the experiment. (*a*) High-viscosity extrusion of crystals embedded in a viscous LCP or HEC matrix results in a cylindrical jet of carrier medium containing crystals. The jet was delivered to the interaction region to intersect with the pump (470 nm laser excitation) and probe (12 keV SwissFEL X-rays) beams used in the experiment. X-ray data were collected at 100 Hz. (*b*) MISP chips with periodic arrays of wells, each containing ideally a single crystal. Mylar foils cap the chip on both sides. (*c*) Non-patterned SOS chips containing crystals in LCP, the same carrier medium as in the HVE jets, but dispensed as a thin flat layer between two Mylar foils. We adjusted the thickness of the LCP layer to match roughly with the crystal dimension. Both patterned MISP and non-patterned SOS chips were rastered in fixed distances and sampled at 100 Hz.

All experiments were performed in air under ambient conditions in the experimental hutch. Three different delivery systems were used: an HVE system designed specifically for the Cristallina-MX instrument, opaque MISP chips and SOS chips, as depicted in Fig. 1 ▸ and Fig. S2. For HVE delivery, a standard MPI 344 µl HVE injector head (Doak *et al.*, in preparation) was clamped into a custom fitting mounted atop a small Standa *XYZ* (8MVT100-251 + 2 × 8MT167S-25) manipulator, which itself was bolted atop the endstation chamber. A small YAG chip was attached to the HVE nozzle, allowing the alignment of the X-rays relative to the pump laser beam to be checked *in situ* during measurement. The microcrystal-containing viscous media were extruded from a 75 µm inner diameter sample capillary at a flow rate of ∼4.2 µl min^−1^. The extruded jet was stabilized using a standard coaxial He gas sheath and then collected on a rotating cylindrical catcher, the rotation speed of which was matched to the jet speed to stabilize the jet further (Doak *et al.*, 2023 ▸). The jet speed was determined by monitoring the Fourier transform of the ‘ladder’ imprinted on the extruded jet by X-rayexposures (Grunbein *et al.*, 2022 ▸). The spacing of the ladder was kept at ∼95 ± 5 µm between successive X-ray pulses, approximately twice the diameter of the pump laser focal spot, by adjusting the jet flow rate.

For experiments using opaque MISP chips, the fixed targets were scanned in the ‘open’ orientation, with the crystal-containing wells facing towards the source of the laser and X-rays, as described previously (Gotthard *et al.*, 2024 ▸). The MISP chips were aligned to the X-rays using fiducial markers using a procedure similar to that of Sherrell *et al.* (2015 ▸), logging the stage positions when the fiducial was at the focus of the on-axis-viewing (OAV) system (∼34 mm from the objective lens). The aperture locations could then be inferred by transforming them onto the surface of this plane. All 26244 wells per chip were sampled at 100 Hz. For SOS chip experiments, each corner of the SOS chip was brought into the focus of the OAV system, and these coordinates were used to define the chip plane. A grid of positions was calculated for each chip and superimposed on the chip plane. A grid spacing of 50 × 50 µm was used for the SOS chips, which is slightly larger than twice the 1/e^2^ diameter of the optical laser pump beam. Both the SOS and MISP chips were scanned in a serpentine-like trajectory using a fast Parker MX80L *XY* stage and a Standa 8MTF *Z* stage.

#### 2.2.2. Photoexcitation

A tunable nanosecond laser (NT230, EKSPLA UAB) was used to photoexcite the FAP microcrystals (Fig. S3). The wavelength of the laser was tuned to 470 nm with an ∼3 ns pulse duration, arriving at the interaction region 500 ns prior to the SwissFEL X-ray probe beam. The intensity stability of the pump laser was measured to have a root-mean-square (r.m.s.) deviation of ∼20% and pump laser intensity data were collected synchronously with the FEL beam during the experiment. An illumination scheme of 6:1 light:dark shots was employed. For the high-viscosity jetting and MISP chip experiments, the optical laser delivery included fiber coupling (105 µm diameter multimode fiber, M15L10, Thorlabs Inc.) between the source and SwissMX endstation, before being focused by the objective of the on-axis microscope (10× M PLAN APO, Optem, Excelitas Technology Corp.) onto the interaction region (Fig. S3). This resulted in a depolarized beam having an approximately flat-top beam profile with a diameter of 52 ± 6 µm (measured by a WinCamD, Data Ray Inc.) and pulse energies (set *via* rotation of a variable optical density filter, NDC-100-4, Thorlabs Inc.) of ∼0.75 µJ or ∼0.15 µJ, corresponding to ∼35 mJ cm^−2^ or ∼7 mJ cm^−2^ (resulting in ∼2.5 and ∼0.5 absorbed photons per chromophore, respectively, assuming an extinction coefficient of ɛ_470nm_ = 11300 *M*^−1^ cm^−1^ and a crystal thickness of 20 µm, and ignoring any potential losses from Fresnel reflections at interfaces).

For SOS chip experiments, the pump beam size was minimized to mitigate light contamination *via* optical scattering, given the completely transparent nature of SOS chips. This was realized by free-space coupling of the laser beam to the on-axis microscope for focusing, which resulted in a tightly focused beam with a Gaussian profile, having a FWHM diameter of ∼6 µm (measured *via* knife-edge scans at the sample position). A quarter wave plate (2-APW-L4-008A, Altechna UAB) was used to circularly polarize the beam to match the beam polarization of the HVE/MISP setup (Fig. S3). Along the beam propagation direction through the depth of the SOS chip, we observed deviations of over half a beam diameter in the spatial beam position from the nominal zero at the SOS chip center, despite the objective being an on-axis focusing system. This is probably due to limitations in tolerances in alignment of the on-axis microscope objective with respect to the X-ray beam trajectory. Extra care in setting and monitoring spatial overlap was necessary given the small beam diameters at the interaction region and noncollinearity between the pump beam and X-rays along the SOS chip depth. These spatial deviations resulted in a variation in fluence at the interaction region by a factor of ∼2.5 between the center and the front and back faces of the SOS chips. Pulse energies of ∼0.1 µJ and ∼0.04 µJ were used in this configuration, corresponding to 244 mJ cm^−2^ and 98 mJ cm^−2^ (∼9 and ∼3.6 absorbed photons per chromophore, assuming, as above, 20 µm thick FAP crystals and no optical losses) at the optimal overlap position. The very high laser fluence was chosen deliberately to test for light contamination, the accidental illumination of nominally dark sample regions, which is a serious concern in transparent fixed targets (Gotthard *et al.*, 2024 ▸).

#### 2.2.3. SFX data collection

SFX data were collected at the beginning of December 2024 using the Cristallina-MX experimental station at SwissFEL using different crystal delivery approaches (Fig. S2). The mean X-ray photon energy was ∼12 keV and the X-ray pulse duration was experimentally determined (Dijkstal *et al.*, 2022 ▸) to be ∼35 fs FWHM. The pulse energy of the attenuated focused XFEL beam at the sample position was ∼100 µJ. Using Kirkpatrick–Baez mirrors, the X-rays were focused to approximately 6 × 6 µm FWHM for data collections using HVE and MISP crystal delivery and to 4.5 × 4.9 µm FWHM for SOS chip crystal delivery. All three experimental setups (HVE, MISP, SOS) were tested by collecting SFX datasets of thaumatin microcrystals (data not shown). These control data were also used to optimize the detector geometry for indexing.

TR-SFX data of FAP microcrystals were collected at 100 Hz with an interleaved pump laser on (light), pump laser off (dark) sequence. In addition, true dark SFX data were collected in the complete absence of pump laser light. Diffraction images were acquired using a JUNGFRAU 8M detector (Dectris, Baden, Switzerland). Diffraction patterns were analyzed using *CrystFEL* (Version 0.10.0), with *peakfinder8* for peak finding, and *xgandalf* and *mosflm* for indexing (White *et al.*, 2016 ▸). Data statistics are shown in Tables 1 ▸ and 2 ▸. Phasing was done by molecular replacement with *PHASER* (McCoy *et al.*, 2007 ▸) using the structure with PDB code 6YRU (Sorigue *et al.*, 2021 ▸) as the search model. The occupancies of the light-induced reaction intermediate were determined essentially as in the work of Barends *et al.* (2024 ▸) and as described in detail in Section 3.1 below. Stochastic errors for these occupancies were estimated by bootstrap resampling as described by Gorel *et al.* (2021 ▸). To investigate any systematic errors due to differences in dataset sizes, the analysis was first performed for all measurements using all of the available indexed lattices/dataset, and then with subsets of data that matched the size of the dataset with the smallest number of images (17000 light lattices, 27000 dark lattices). The images for these subsets were chosen randomly (see also the legend to Table 3 ▸).

## 3. Results and discussion

SFX pump–probe experiments can yield unprecedented insight into reaction mechanisms, in particular on the ultrafast timescale. However, detecting the evolving structural changes can be highly challenging. In general, the faster the process, the smaller the changes and thus the signal. This is compounded by the fact that typically only a fraction of the molecules in the crystal actually react. To boost the signal-to-noise ratio of features in difference electron-density maps, a common approach in the community is to increase the pump laser energy. Concerns about multiphoton excitation, often changing the photophysics and/or photochemistry and thus the reaction coordinate, as well as the dynamics of the structural response [see for example Barends *et al.* (2024 ▸)], have been swept aside using the argument that a very large fraction of the incident photons are scattered by the crystal-delivering jet, never interacting with the light-sensitive molecules. This is a serious and so far highly controversially discussed issue, affecting not only the mechanistic interpretation of published data but also the design of future experiments.

To assess whether there are significant losses in pump laser fluence in viscous jet-based TR-SFX measurements, we performed TR-SFX experiments on FAP crystals illuminated with two different pump laser intensities using high-viscosity jets, fixed-target MISP chips and non-patterned SOS chips for sample delivery on the Cristallina-MX instrument at SwissFEL. FAP is ideally suited for this investigation as it has a high quantum yield (∼80%) and undergoes an irreversible photoinduced de­carboxyl­ation reaction, forming a transient intermediate that involves large structural changes of the fatty acid substrate (removal of carboxyl­ate group, movement of the alkyl tail) that are straightforward to detect (Sorigue *et al.*, 2021 ▸). The photoproduct yields were then determined to quantify the effects of the sample delivery method on the effective pump laser fluence reaching the crystals. Reliable and accurate occupancy determination is thus essential for the outcome of this (and any other) TR-SFX experiment.

### 3.1. Occupancy determination

The determination of the occupancies of the various species in photolyzed (or otherwise excited/triggered) crystals is not trivial. In pump–probe diffraction experiments, one typically does not reach 100% occupancy of the light- (or whatever trigger was used) induced state, but an unknown mixture of not-triggered and triggered states. Typically, one assumes that there is only a single structurally definable triggered state. This state’s electron density is usually calculated from *extrapolated structure factors* (Genick *et al.*, 1997 ▸) derived from data collected from dark-state crystals as well as crystals exposed to light (or whatever triggering method was used). These extrapolated structure factors are estimates of the amplitudes that would have been measured if the triggered state had been present at 100% occupancy, and they can be used to determine the structure of that state. Crucially, the extrapolation process requires the determination of an assumed ‘occupancy’ of the excited state, and various methods have been proposed to determine its optimal value (Genick *et al.*, 1997 ▸; De Zitter *et al.*, 2022 ▸; Barends *et al.*, 2024 ▸). However, this number can underestimate the actual physical occupancy by a large amount, as much as a factor of two (Barends *et al.*, 2024 ▸; Schmidt, 2023 ▸). One could also, in principle, use occupancy refinement during the refinement of an ensemble model of both dark and light states to determine the true physical occupancy. However, occupancy refinement is often highly unstable, as occupancy is strongly coupled to atomic *B*factors.

**Figure 2:**
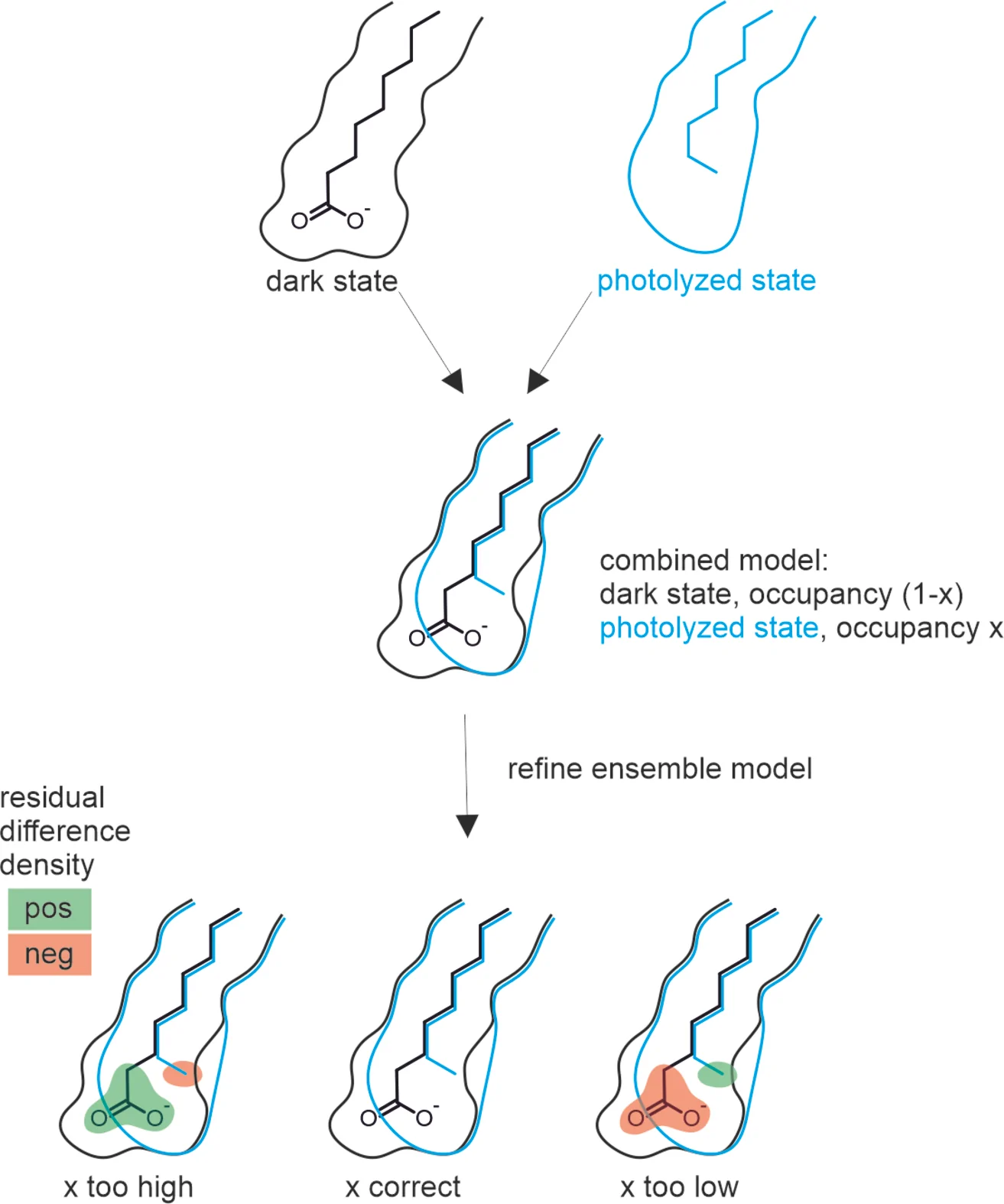
Determination of phototriggered-state occupancy *x* by ensemble refinement. A model of the dark state, obtained from refinement against pure dark-state data, is combined with a model of the pure photolyzed state obtained using extrapolated structure factors. In this ensemble model, the occupancy of the photolyzed state is set to *x* and the occupancy of the dark state to (1 − *x*). Such ensemble models are prepared using a range of values for *x* and each model is refined against data from partially photolyzed crystals. The residual difference density in the active site is used to evaluate whether the chosen value for *x* is correct. For instance, negative difference density at the position of the substrate carboxyl­ate indicates that the value of *x* is too low, whereas positive density indicates that the value of *x* is too high. If the value of *x* is chosen correctly, no residual density should be present.

**Figure 3:**
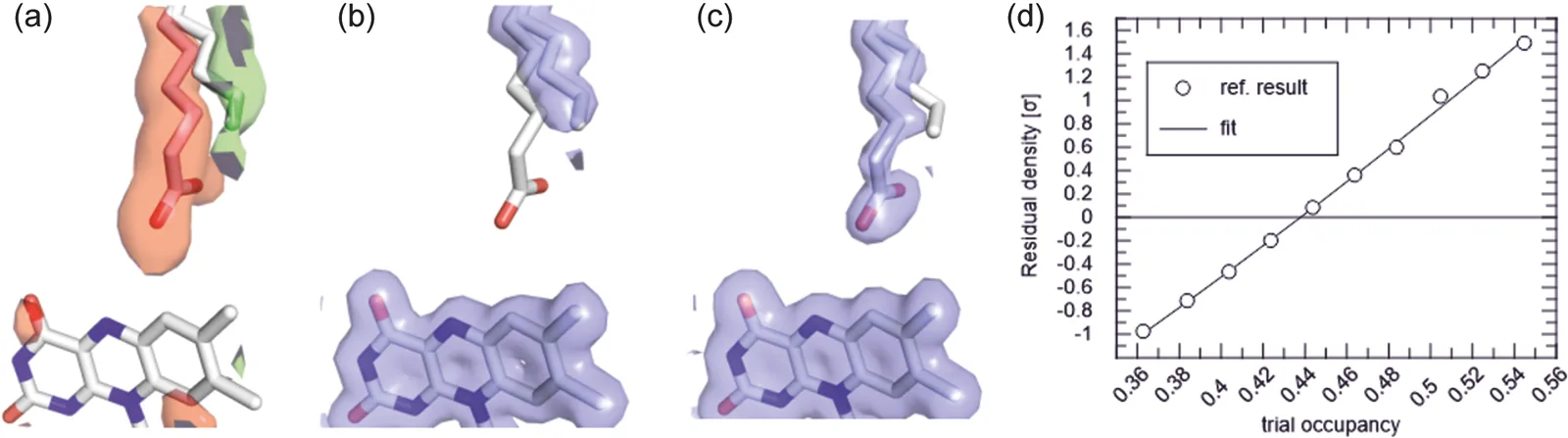
Electron-density maps during light-state occupancy determination. The substrate, product and FAD cofactor are shown as sticks. (*a*) Light–dark difference density map, contoured at ±3.0σ (green/red). The de­carboxyl­ation of the fatty acid substrate and the change in conformation of the product alkyl chain are clearly visible. (*b*) Extrapolated electron-density map, contoured at 1.0σ, showing density for the photolyzed state only. (*c*) 2*mF*_o_ − *DF*_c_ electron-density map contoured at 0.8σ after ensemble refinement. The contribution of the photolyzed state (occupancy ∼0.44) can just be made out in the density of the alkyl chain of the product. (*d*) Plot of the residual density at the carboxyl­ate position for various assumed occupancies during occupancy determination. Circles show the data points and the straight line is fitted through these points. A clear zero-point crossing is observed at ∼0.44. Note that this graph shows the second of two searches for the zero-point crossing; initially, a much coarser search in terms of trial occupancies (0.05–0.9) is performed, leading to much higher absolute difference map values. The second, finer, search is performed around the zero-point crossing found in the initial, coarse, search. However, the results of the coarse and fine searches typically do not vary by more than 0.03 in occupancy.

We have recently developed another method based on ensemble refinement for occupancy determination (Barends *et al.*, 2024 ▸). In this method (Fig. 2 ▸), called occupancy scanning ensemble refinement, extrapolated structure factors (Genick *et al.*, 1997 ▸) are first used to produce the best possible model of the photo-triggered state. As stated above, while the ‘occupancy’ determined in the extrapolation process does not, in all likelihood, represent the true physical occupancy, the structure of the photo-triggered state can be determined accurately in this way. To determine the true physical occupancy, this model is then combined with a model of the dark state, assuming a certain true occupancy. This ensemble model is then refined against data collected from the light-exposed (or otherwise excited) crystals, monitoring the residual difference electron density at a position in the structure where a large structural change occurs upon excitation. This is then done for a range of possible occupancies. At the correct occupancy, there should be no residual difference electron density after refinement (Fig. 3 ▸). We previously validated this method for the carb­oxy­myoglobin system using synthetic data (Barends *et al.*, 2024 ▸) and repeated this validation for the current FAP data (see Appendix *A*[App appa]). In this case, de­carboxyl­ation of the fatty acid substrate and movement of the generated alkane chain results in negative electron density around the carboxyl group, C2 and C3, of the fatty acid and positive electron density for the newly positioned alkane chain (Fig. 3 ▸). For most samples, the value of the difference density at the position of the C2 atom was used for occupancy determination. For HEC-embedded samples, a position between the C1 and C2 atoms was used because of an interfering density peak close to the C2 atom.

### 3.2. Comparison of the photoproduct yield using MISP and HVE crystal delivery

When comparing photoexcitation yields using different experimental setups, it is important to use the same crystals and photoexcitation conditions. The photoexcitation fluence must be low enough to ensure linearity of the photoproduct yield with the number of absorbed photons. Previous power titration studies (Sorigue *et al.*, 2021 ▸) using 2.7 ps excitation pulses showed an ∼3 ps rise component of the *S*1→*S*0 emission under multiphoton conditions (∼50 mJ cm^−2^, 2.3 absorbed photons), which occurs instantaneously at sub-stoichiometric excitation (*e.g.* ∼4 mJ cm^−2^ or ∼9 mJ cm^−2^, corresponding to ∼0.2 and 0.4 absorbed photons, respectively). These processes occur on a much shorter timescale than the ∼300 ps electron transfer (ET) time, which appears insensitive to the excitation power. A small difference in the shape of the 1 ns spectra (long-lived component) at 50 mJ cm^−2^ versus the lower fluence can be assigned to a fraction of long-lived FAD* molecules (where FAD is flavin adenine dinucleotide, the chromophore in FAP) due to substrate depletion at very high excitation power. No evidence was found for further flavin products. Thus, in the case of FAP, multiphoton excitation does not seem to result in side products, but merely in a loss of linearity between fluence and photoproduct yield.

We tested both low and high laser fluences (corresponding to nominally ∼0.5 and ∼2.5 absorbed photons per chromophore on average, when assuming a crystal thickness of 20 µm and the absence of any scattering) in MISP chips and HVE jet delivery experiments. The lower fluence is in the linear excitation regime, so as to be able to correlate the number of absorbed photons with the photoproduct yield. The higher laser fluence was chosen for two reasons: First, to ensure observation of photoproduct signal in case the published high scattering losses turned out to be real, and second, to test for light contamination, *i.e.* the accidental illumination of neighboring crystals. Such contamination is a result of undesired pump light scattering and can severely complicate the interpretation of experimental observations. To test for light contamination we collected interleaved dark data during the pump–probe illumination sequence; in the case of light contamination, difference electron-density maps calculated from interleaved dark data and data collected in the absence of any pump laser (true dark) would show photolysis features. However, because our interleaved dark datasets contain about six times fewer images than their respective light datasets, the diffraction amplitudes derived from them will have higher error levels, meaning that in maps calculated from them photolysis features will be weaker and noisier than in maps computed from larger numbers of images. We therefore first estimated the minimum amount of light contamination detectable with our limited interleaved dark data, using two methods. First, we repeated the validation of our occupancy determination method (Appendix *A*[App appa]) using the higher error levels obtained with the interleaved dark data and found that, under the conditions used, the first signs of light contamination (*i.e.* difference map features caused by photolysis) started to become visible at 10% occupancy. Second, we prepared various synthetic datasets by mixing images from light-exposed data with images from dedicated dark data in various proportions to simulate data with various levels of light contamination and similar numbers of images as in our interleaved dark data sets. Here, too, difference map features indicating photolysis were visible from 10% occupancy onwards. We then finally calculated maps using the actual interleaved dark data and found these to be featureless (Fig. S4), indicating that the data contain at most 10% of the light-state occupancy.

Patterned fixed targets, such as the MISP chip (Carrillo *et al.*, 2023 ▸), contain hard-patterned arrays of crystal localization wells that form physical barriers between and around crystals that can prevent accidental illumination of the sample by scattered pump light when using opaque chip materials (Gotthard *et al.*, 2024 ▸). The crystals are funneled into the wells when removing the crystal storage solution *via* the through holes by vacuum suction or blotting, leaving only minimal amounts of liquid around the crystals that could scatter optical pump photons. Other scattering sources (apart from the crystals themselves) are the funnels and holes containing the crystals and the two Mylar foils encasing the chip. Unlike the SOS chips which have a built-in mechanism for stretching the Mylar foils taut (Doak *et al.*, 2024 ▸), the current implementation of the MISP chips does not provide for reproducible stretching of the Mylar foils covering the chip. Sometimes they are rather loose, bulging out or clinging to the chip surface, and sometimes they are taut (Fig. S5). This affects the thickness of the solvent film that can form between the chip and the Mylar foil. The amount of liquid around the crystals depends on a number of parameters, including the ‘pulling force’ of the vacuum applied to remove the mother liquor. Since FAP crystals are mechanically sensitive and shatter very easily (as indicated by a large number of images containing diffraction patterns from multiple crystals, independent of the initial crystal concentration used for loading the chips), we opted for relatively wet crystals. Depending on the amount of liquid around the crystals, small vapor droplets occasionally formed on the Mylar foil facing the pump laser beam (Fig. S5). All of these effects can result in pump laser scattering. Therefore, we collected datasets that differed not only in the pump laser energy (low and high) but also in the exit aperture size used, as this also affects the amount of liquid remaining in the setup. No significant difference in photoproduct yield was observed for the two apertures tested (squares of side length 6 ± 1 µm and 10 ± 2.5 µm, respectively; Table 3 ▸) using either all available data or with 17000/27000 lattices. Thus, we have high confidence in the derived photoproduct yields of ∼45% and ∼23% for the high and low pump laser energy densities, respectively (using all data, see Table 3 ▸).

HVE jets were developed to enable SFX investigations using crystals of membrane proteins grown in LCP (Weierstall *et al.*, 2014 ▸). The low adjustable speed of viscous jets can be easily matched to an XFEL repetition rate of 30–120 Hz, allowing for efficient sample usage, particularly compared with high-speed liquid-jet injection using gas dynamic virtual nozzles (GDVNs; Weierstall *et al.*, 2012 ▸). This also makes HVE highly attractive for delivering microcrystals of soluble proteins into the XFEL beam. In this case LCP can be used for embedding, but other media, cheaper and easier to handle, are also possible, such as HEC (Sugahara *et al.*, 2017 ▸). Both LCP and HEC are optically transparent, making them highly suitable for use for pump–probe experiments (Niziński *et al.*, 2025 ▸).

Crystal-containing jets are inherently inhomogeneous materials, which often leads to unstable jetting behavior. Thus, viscous jets often display unstable speeds and are prone to positional instability, even when using gas focusing. In TR-SFX pump–probe experiments this behavior can result in light contamination, specifically when the jet speed is too low and the illuminated material has not cleared the interaction region before the subsequent pump pulse arrives. We mitigated possible light contamination by adjusting the speed of the HVE jets such that the displacement between X-ray shots was approximately twice the diameter of the pump laser beam (∼95 ± 5 µm per shot). Two datasets each were collected for high and low pump laser energy densities, with crystals embedded in LCP for HVE delivery. The photoproduct occupancies of both datasets are comparable with those derived from the MISP data (Table 3 ▸). In particular, the photoproduct occupancy using MISP chips for crystal delivery is highly similar to the occupancies observed when using HVE of LCP-embedded crystals (Table 3 ▸) when photoexciting in the linear regime (7 mJ cm^−2^). This implies that neither the cylindrical jet geometry nor the additional LCP matrix induces significant scattering that would result in appreciable loss of photoexciting photons.

**Figure 4:**
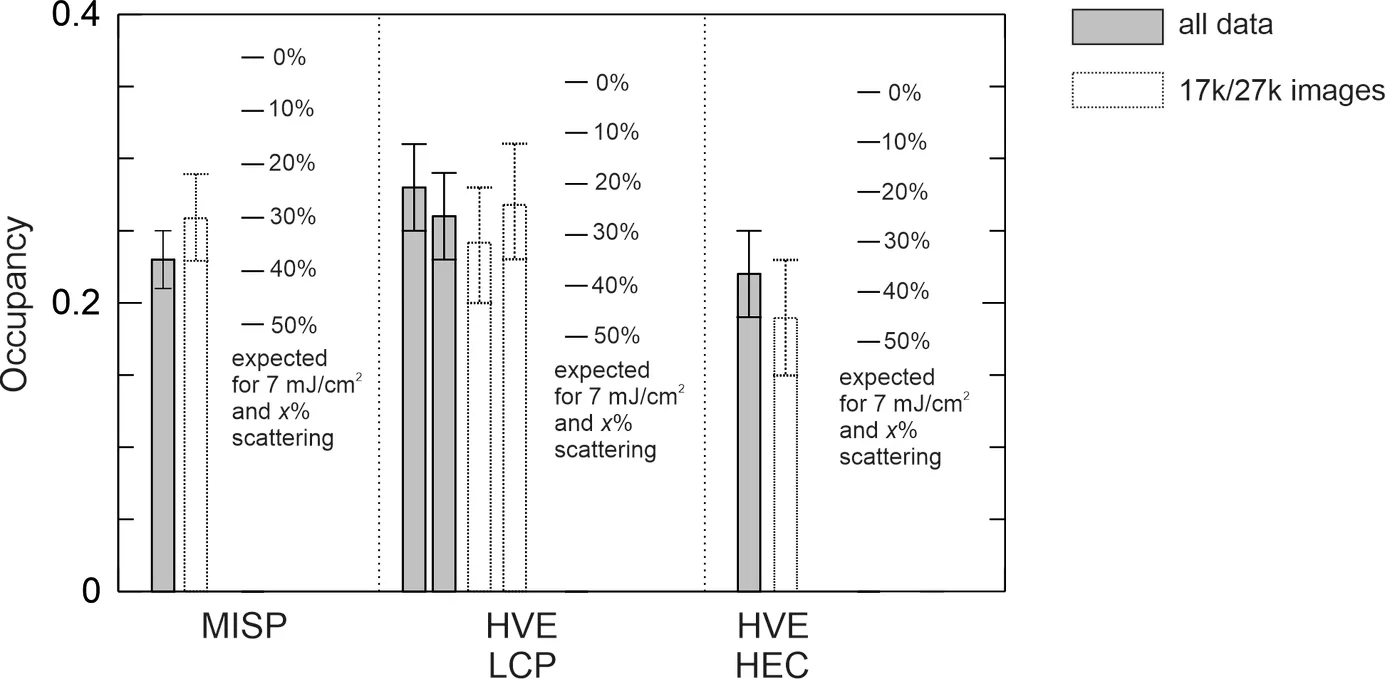
Comparison of experimentally determined light-state occupancies (gray columns) with expected occupancies for various levels of pump light loss by scattering and Fresnel reflections (numbers on the right). The dashed data points show the occupancies derived from datasets with reduced numbers of images, but the best estimates of the occupancies and their errors are those determined using all data.

HEC is an optically transparent medium that is commonly used for crystal embedding for HVE injection, since it is much cheaper and typically also easier to prepare than LCP. Therefore, we also embedded FAP microcrystals in HEC. Compared with LCP, HEC is relatively stiff and therefore has a higher propensity to form a straight jet. While this property is experimentally advantageous, it is problematic for mechanically sensitive samples such as FAP crystals. Mixing the crystals into ∼14% HEC results in significant degradation and the crystals shatter into small crystallites (Fig. S1). This very clearly shows that HEC is not a suitable embedding material for FAP crystals, emphasizing the necessity for a thorough investigation of the suitability/compatibility of the matrix material and investigated crystals (see the supporting information). For a ‘real’ mechanistic investigation, we would not have pursued HEC embedding any further, but for the sake of completeness of the current technical investigation we include the data here. Despite the strong degradation of the HEC-embedded FAP crystals, they still diffract to a resolution of ∼2.1 Å (for LCP ∼1.7 Å). The photoproduct occupancy (Table 3 ▸, Fig. 4 ▸) appears to be somewhat lower than for crystals embedded in LCP. While the weaker, lower multiplicity and lower resolution diffraction data could affect the accuracy of the occupancy determination (in particular for the low fluence photoexcitation), the reason for the apparent discrepancy is unclear. It is conceivable that the mechanical properties (*i.e.* the stiffness) or dehydration of the embedding medium influence the protein dynamics. Clearly, close attention needs to be paid when choosing an embedding medium for the crystalline system investigated. This concerns not only their mechano/chemical compatibility, which can affect the external appearance (Fig. S1) and/or internal order [resolution, mosaicity, unit-cell distribution (Tables 1 ▸ and 2 ▸), structural changes due to dehydration] of the crystals, but also the optical properties of the embedding material. It should be optically transparent, not only before embedding the crystals but also afterwards. Potential issues include metal debris from the mixing syringes (Niziński *et al.*, 2025 ▸) or phase separation, which is particularly problematic for hydro­phobic matrices such as Super Lube. In the latter case, even very small amounts of aqueous liquid surrounding the crystals can result in significant scattering (Niziński *et al.*, 2025 ▸).

### 3.3. SOS chips for pump–probe experiments

SOS fixed-target chips are a versatile, cheap and easy to use crystal delivery method for serial crystallography data collection at synchrotrons and XFELs (Doak *et al.*, 2024 ▸; Gorel *et al.*, 2025 ▸). Their defining feature is the absence of wells housing microcrystals, which removes limitations on crystal size and embedding medium. This enables, for example, the investigation of membrane protein crystals grown in LCP in non-patterned fixed-target chips, a significant challenge for patterned chips. However, the absence of patterned physical restrictions is also a weakness of the SOS chips when it comes to avoiding light contamination of adjacent crystals. To mitigate this problem, we focused the pump laser as tightly as possible [∼6 × 6 µm (FWHM)]. To test whether this approach indeed avoids light contamination, we exposed the FAP microcrystals in SOS chips to an excessively high laser fluence (∼245 mJ cm^−2^, corresponding to ∼9 photons per chromophore, and ∼97 mJ cm^−2^, corresponding to ∼3.6 photons per chromophore) to detect even small fractions of scattered light resulting in light contamination. Diffraction data were collected using translational spacings of 50 µm, corresponding to more than twice the 1/e^2^ diameter of the pump beam and similar to the illumination distance chosen in the viscous jets. Achieving and maintaining overlap of such a tightly focused optical laser with the X-ray beam at the interaction region is particularly challenging for this setup, where the observed noncollinearity and narrow depth of field of the focused beam will necessarily result in discrepancies in spatial overlap and pump laser fluence throughout the depth of the SOS chip (Fig. S3).

We observed no light contamination in the SOS datasets collected with 50 µm spacing, despite the excessively high pump laser fluence. This absence of light contamination and the observation of significant photoactivation (occupancy of ∼0.4, Table 3 ▸) for FAP crystals illuminated with an excitation fluence of ∼97 mJ cm^−2^ (3.6 absorbed photons per chromophore) shows that SOS chips can be used successfully for pump–probe investigations and studies of membrane protein crystals grown in LCP. Obviously, for meaningful experiments investigating photoproducts (and not mainly scattering as in the current article), much lower laser fluences should be used to prevent saturation effects, side reactions and local sample heating.

### 3.4. Comparison of absolute photoproduct yields

The comparison of the expected photoproduct yield and the experimentally derived absolute occupancies of FAP photoproducts in crystals delivered by HVE jet and MISP chips also yields important information regarding the magnitude of possible losses in optical pump fluence due to the sample delivery environments.

Photoproduct yields can be predicted from the reaction quantum yield and the pump light absorption. Naïvely, one might think this latter quantity can be calculated using the Beer–Lambert law, *i.e.* by multiplying the crystal thickness with the concentration and molar absorption coefficient of the chromophore. However, molar absorption coefficients are almost invariably determined in solution, where the chromophores are randomly oriented. The probability that a chromophore absorbs a photon depends on the orientation of the chromophore with respect to the polarization of the incoming light, but these values do not apply to crystals, where the chromophores are not randomly oriented. Specifically, for circularly polarized light, the absorption probability is proportional to sin^2^α, where α is the angle between the chromophore’s transition dipole moment and the electric field vector of the light. Macroscopically, the relevant quantity is the average over all chromophores, 〈sin^2^α〉, which for circularly polarized light and a perfectly random distribution of chromophore orientations as in solution is 0.66666…. In a crystal, however, this distribution is not random and the absorption can accordingly be much weaker or much stronger than would be expected from the solution absorption coefficient, depending on how the crystal and the chromophores inside it are oriented (Ng *et al.*, 1995 ▸). Importantly, however, the orientations from all crystals contributing to the dataset can be extracted from the crystallographic data. Since the direction of the transition dipole of FAD in the asymmetric unit is known, one can determine the direction of the transition dipoles of the various FAD molecules in the unit cell from the FAP crystal structure by applying the space group’s symmetry operators. By applying the orientation matrices of the crystals contributing to a dataset, the distribution of all transition dipole orientations can thus be determined. Combining this information with the geometry of photoexcitation (in particular the pump beam direction), one can calculate 〈sin^2^α〉 for each of the sample presentation methods tested. For the MISP, LCP and HEC data the values of 〈sin^2^α〉 are ∼0.68, ∼0.63 and ∼0.61, respectively. These values can be used to correct the solution molar absorption coefficient for the effects of nonrandom chromophore orientation (Appendix *B*[App appb]).

In addition, one needs to take into account the fall off in the intensity of the pump light as it travels through the crystal, which can be done using rate equations describing the accumulation of the converted molecules along the path of the light. We derive a simple set of formulae for this in Appendix *B*[App appb]. When combined with the orientation effects discussed above, the final values for the expected fractional photoproduct yields, and therefore for the expected crystallographic occupancies of the light state for the MISP, LCP and HEC 7 mJ cm^−2^ data (*i.e.* using 0.5 photons per chromophore), are 0.37, 0.35 and 0.35, respectively. Thus, when considering only the full data sets, for the MISP data 62% of the expected photoproduct yield was reached in the experiment, whereas for LCP 74–79% of the expected photoproduct yield was obtained. For HEC, 64% of the expected occupancy was reached (Table 3 ▸).

Importantly, the occupancies reported here have error levels of up to ±0.05 as determined by bootstrap resampling. In relative terms, this corresponds to as much as 20% of the actual value for some data points. At these error levels, even differences in apparent occupancy that might seem large are statistically not significant; for instance, for HVE LCP 1 and 2 data (using all images) the occupancies found are 0.44 and 0.40. It might be tempting to try to explain the 10% relative difference between the two values by some experimental factor, but this corresponds to just a 1σ difference and is statistically insignificant. The same holds for other cases such as the high fluence SOS (50 µm) data, where there is a difference of 0.07 (as much as 18% of the numerical value) between the values obtained with all data and with limited data (17000/27000 images). Here, too, given the error estimates obtained by bootstrapping, these seemingly large differences are not significant. Indeed, a systematic study of the influence of the number of images on the apparent occupancy shows that the number of images adds another degree of variability, reducing the precision beyond what bootstrapping on a single dataset alone suggests. However, given that the inclusion of more images in a serial crystallography dataset generally improves data quality, the occupancy values derived from the full datasets are likely to approximate the true values more closely and will therefore be used to assess the magnitude of any scattering effects.

In an earlier study, we reported on error levels in occupancies determined by extrapolation and by comparing difference map peaks (Barends *et al.*, 2024 ▸) and found similar absolute errors of several percentage points at comparable occupancies to those found here, despite high-quality 1.5 Å resolution data. It would appear, therefore, that with the methods currently available, the occupancies of light-induced states in pump–probe crystallography are inherently difficult to determine precisely. What consequences this has for the final light-state structures, the successful determination of which depends on knowing their occupancy, will be the subject of further study. Nevertheless, this raises the question of what levels of scattering our experiments could have detected.

Importantly, to estimate this, only the results at 0.5 photons per chromophore should be used, since at 2.5 photons per chromophore the photoproduct yield is already close to saturation. Fig. 4 ▸ shows a comparison of the occupancies found experimentally with the expected photoproduct yields considering various amounts of scattering, assuming that scattering is the only cause of the observed discrepancy. This suggests that, given the error levels in our experimentally determined occupancies, the levels of pump light scattering during the experiments were around 35–45% for the MISP data, 10–35% for the LCP data and 25–45% for the HEC data. However, these scattering losses are probably overestimated since they also include losses due to Fresnel reflections at the air/jet or air/chip interface and additional Fresnel reflection at the crystal surface, which has been estimated to amount to an approximately 10–20% reduction in the intensity of the pump beam [see also Grünbein *et al.* (2020 ▸)]. Moreover, they do not take into account poorly quantifiable losses such as through scattering off condensed vapor droplets as in the MISP chip (Fig. S5).

However, effects other than scattering could also contribute to the discrepancy between expected photoproduct yields and experimental occupancies. Consider, for instance, a hypo­thetical case in which all dipoles in a crystal are exactly collinear. In such a case, the crystal acts as a polarizer and, no matter how strong the absorption is, the light component perpendicular to the dipoles will always go through without absorption [see ch. 10.9 of Lakowicz (2006 ▸)]. Since the transition dipole moments in the FAP expanded unit cell are almost collinear, this means that light propagating deep into the crystal will be less absorbed, reducing photoproduct yield beyond what is expected using our simple formulae. A more precise assessment of this effect exceeds the scope of this work, however.

Nevertheless, the discrepancies between experimental and expected occupancies set a clear upper limit for the magnitude of pump light losses through scattering of 20–40%.

## 4. Conclusions

Within the limits of our method, comparison of the calculated photoproduct yields and experimentally derived occupancies for all three sample delivery methods used suggests losses of the order of 20–40%. Despite initial assumptions that the fixed-pattern chips represent an idealized sample delivery platform which should largely mitigate optical losses, we observe considerable variability between the high and low fluence data compared with HVE LCP jets, and a significant discrepancy between expected and recovered occupancy at low fluence, which point to the complexities of this sample delivery approach. In contrast, HVE LCP jets showed the minimum of occupancy losses for low fluence excitation and a simple accounting for lossy Fresnel reflections (∼10%). Scattering losses in the viscous matrix [see Niziński *et al.* (2025 ▸)], estimated between 0–30%, combined with the gains expected from the focusing effects of the cylindrical jet in the horizontal plane (Grünbein *et al.*, 2020 ▸), lead to a near-quantitative understanding of the excitation conditions in HVE delivery with LCP. Even so, we wish to highlight the care needed to quantify occupancy accurately in even well understood systems such as FAP and that this remains a challenging task in the best of cases. Still, these observations definitively refute the notion that an overwhelming fraction of the optical pump beam fails to reach the interaction region in optically transparent viscous jets and show that for correctly sized crystals of molecular systems with high quantum yields, physically sensible occupancies are readily recovered.

Consequently, it is highly likely that previously published TR-SFX experiments using femtosecond pump pulses (Barends *et al.*, 2015 ▸; Nogly *et al.*, 2018 ▸; Claesson *et al.*, 2020 ▸; Carrillo *et al.*, 2021 ▸; Dods *et al.*, 2021 ▸; Mous *et al.*, 2022 ▸; Gruhl *et al.*, 2023 ▸; Christou *et al.*, 2023 ▸; Maestre-Reyna *et al.*, 2023 ▸; Yun *et al.*, 2021 ▸; Cellini *et al.*, 2024 ▸; Shankar *et al.*, 2025 ▸; Malla *et al.*, 2025 ▸) were performed under multiphoton excitation conditions. It is unclear whether any erroneous conclusions have been drawn based on multiphoton effects. For future experiments, however, there is no justification for choosing to use excessive pump laser fluence in the low scattering environments provided by optically transparent carrier media, nor for crystal delivery in fixed-target chips (see the supporting information). Vigilance is required when choosing the viscous matrix for HVE injection [see also Niziński *et al.* (2025 ▸)] and crystal sizes (Grünbein *et al.*, 2020 ▸) and, to avoid unpleasant surprises, crystal properties and sample delivery need to be matched carefully.

## 5. Related literature

For further literature related to the supporting information, see Grünbein & Nass Kovacs (2019 ▸), Hutchison *et al.* (2016 ▸), Li *et al.* (2021 ▸), Martiel *et al.* (2019 ▸), Sugahara *et al.* (2015 ▸), Sugahara *et al.* (2016 ▸), Sugahara *et al.* (2020 ▸) and Zarrine-Afsar *et al.* (2012 ▸).

## Supplementary Material

Extended discussion and additional figures. DOI: 10.1107/S2052252526005403/zf5028sup1.pdf

## Figures and Tables

**Figure 5:**
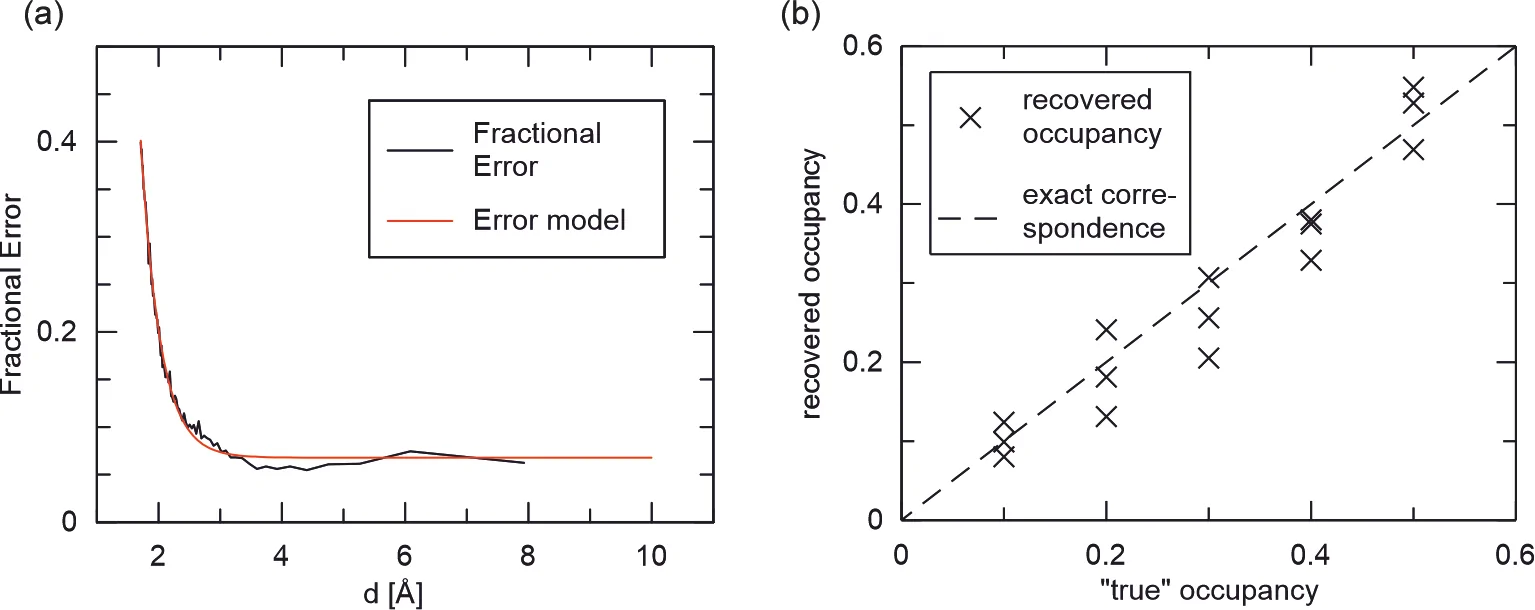
(*a*) Error model derivation for the calculation of realistic test data. The average fractional error of a real dataset was plotted as a function of resolution (black line). An error model was produced by fitting an exponential function to this fall off. The error model was used to calculate realistic test data as described in the text. (*b*) Validation of the occupancy retrieval method. Calculated test data with realistic random errors were produced for a range of photolyzed state occupancies (in triplicates). These data were subjected to the occupancy retrieval method described in the text and the retrieved occupancy is plotted as a function of the actual occupancy used to produce the test data. The method retrieves the ‘true’ occupancy within 5–7 percentage points in most cases.

**Figure 6:**
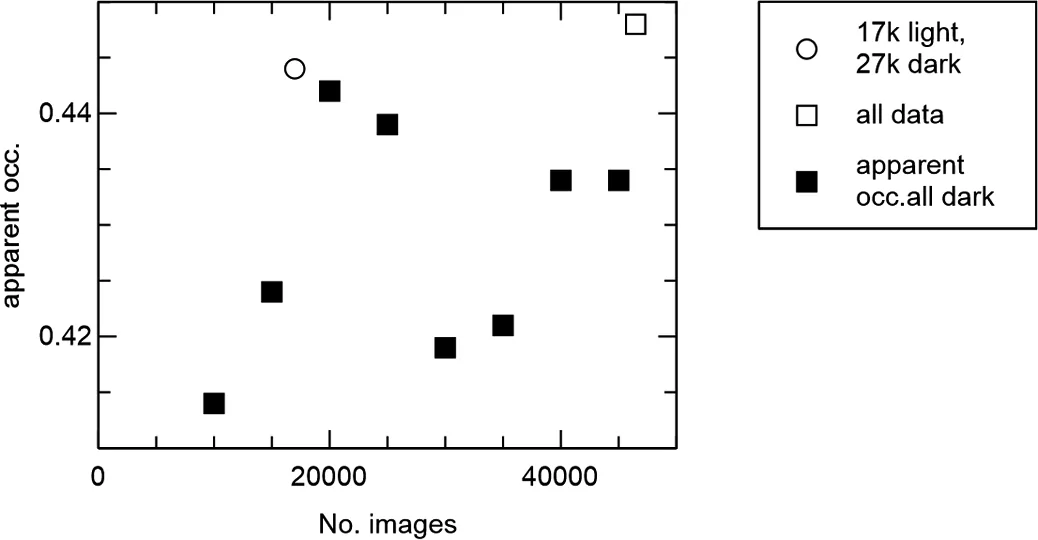
Effect of number of images in the light dataset on the occupancy determined by ensemble refinement. The values reported in Table 3 ▸ are shown with open symbols.

**Figure 7:**
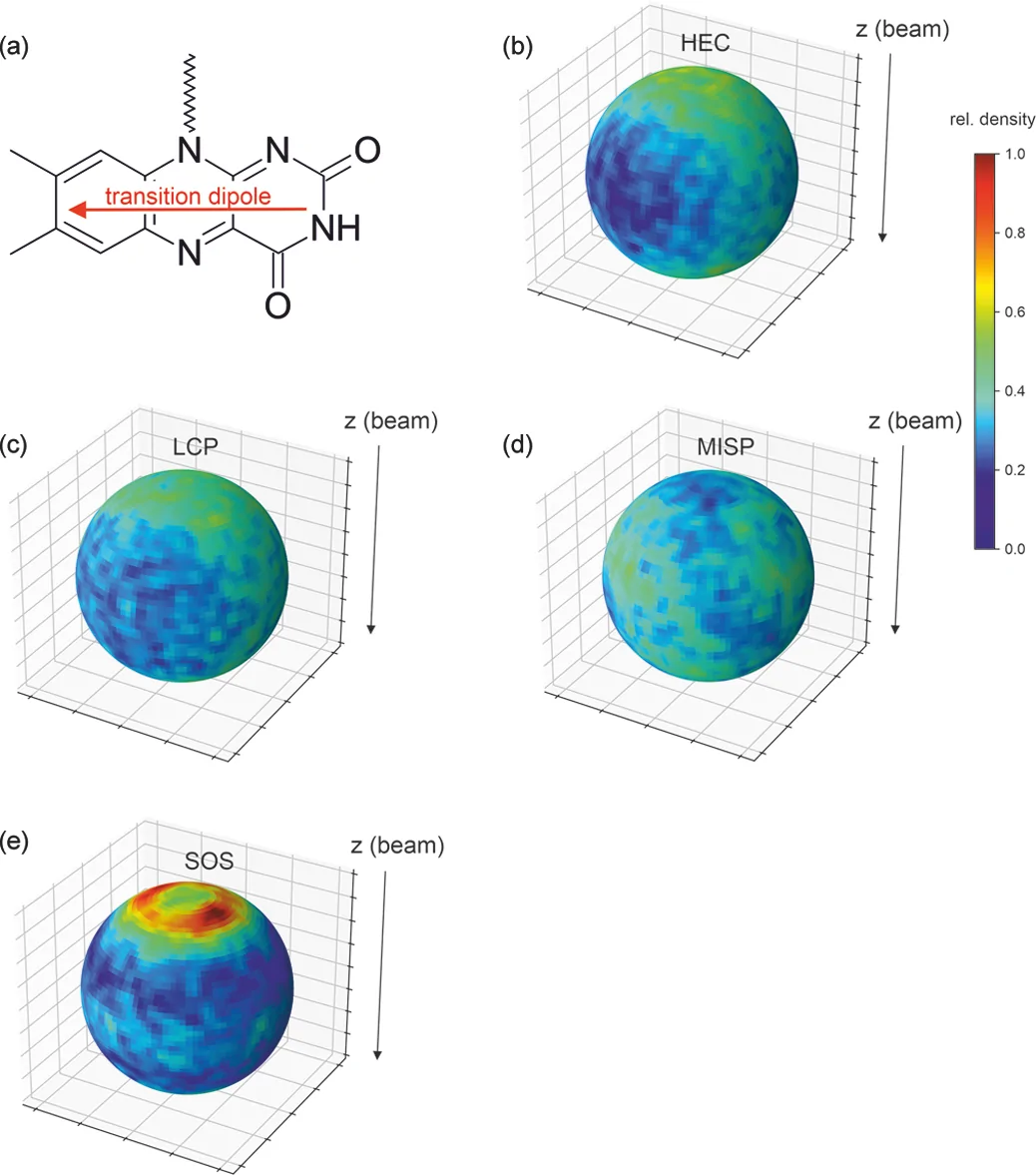
(*a*) Orientation of the FAD transition dipole. (*b*), (*c*), (*d*) and (*e*) Distributions of transition dipoles for the HVE [using (*b*) HEC and (*c*) LPC crystal embedding matrices], (*d*) MISP and (*e*) SOS data. Each data set contained 17000 lattices. As there are eight molecules of FAP per unit cell, the distributions shown here contain 136000 transition dipoles each. Note that the calculation of 〈sin^2^α〉 for the SOS case is complicated by the fact that the pump laser direction is not collinear with the *Z* direction.

**Figure 8:**
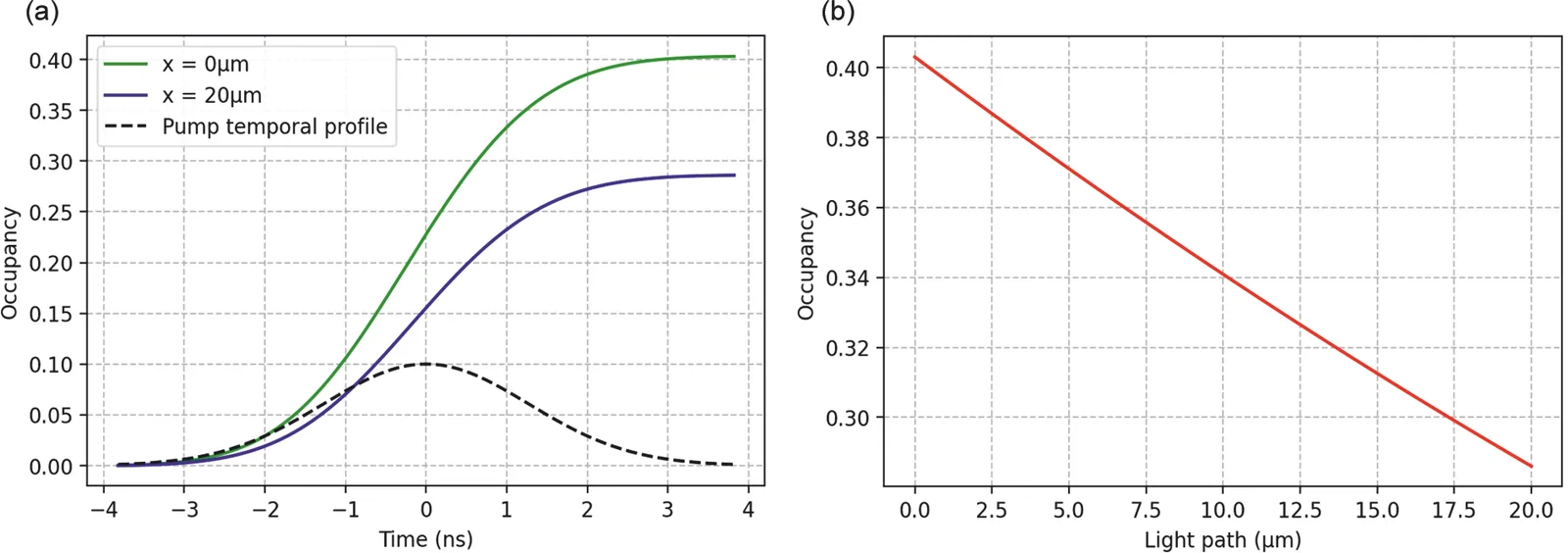
(*a*) Fraction of the photoactivated states accumulated during the nanosecond pulse (470 nm, 7 mJ cm^−2^) for the front and back faces of the 20 µm thick crystal. (*b*) Fraction of the photoactivated states accumulated after the pulse along the light path in the crystal. The average of the red line is 0.34, representing the expected occupancy. This value was obtained using equation (10), assuming sin^2^α = 〈sin^2^α〉 = 0.6, ɛ = 11300 *M*^−1^ cm^−1^, Φ = 0.8 and *c* = 0.0091 *M* (note that *N* = 10^−3^*N*_A_*c*).

**Table:** ‘High’ or ‘low’ in the dataset name indicates the pump laser energy, see Table 3. Values in parentheses are for the high resolution shell data.

	Dataset					
	HVE (LCP)1 high	HVE (LCP)1 low	HVE (LCP)2 high	HVE (LCP)2 low	HVE (HEC) high	HVE (HEC) low
Space group	*I*222	*I*222	*I*222	*I*222	*I*222	*I*222
Unit-cell parameters (Å, °)	93.8, 104.9, 158.6, 90.0, 90.0, 90.0	93.6, 104.8, 158.5, 90.0, 90.0, 90.0	93.8, 105.0, 158.6, 90.0, 90.0, 90.0	93.8, 105.0, 158.6, 90.0, 90.0, 90.0	94.4, 105.2, 157.9, 90.0, 90.0, 90.0	94.5, 105.2, 158.0, 90.0, 90.0, 90.0
No. of indexed lattices	37729	38240	38446	42115	38728	38229
Resolution limits (Å)	10.00–1.67 (1.71–1.67)	10.00–1.67 (1.71–1.67)	10.00–1.63 (1.67–1.63)	10.00–1.60 (1.64–1.60)	10.00–2.00 (2.05–2.00)	10.00–1.92 (1.97–1.92)
*I*/σ(*I*)	5.5 (1.1)	5.8 (1.2)	5.9 (1.2)	5.9 (1.1)	4.6 (1.3)	4.2 (1.1)
*R*_split_ (%)	13.2 (96.5)	12.8 (89.4)	12.6 (88.8)	12.0 (102)	16.5 (82.8)	17.6 (105)
CC^1/2^	0.976 (0.492)	0.974 (0.519)	0.975 (0.558)	0.979 (0.438)	0.971 (0.533)	0.97 (0.382)
CC*	0.994 (0.812)	0.993 (0.827)	0.994 (0.846)	0.995 (0.78)	0.993 (0.834)	0.992 (0.744)
Completeness (%)	100.0 (100.0)	100.0 (100.0)	100.0 (100.0)	100.0 (100.0)	100.0 (100.0)	100.0 (100.0)
Multiplicity	414.3 (260.4)	436.9 (274.3)	332.2 (204.8)	354.3 (235.3)	198.6 (143.3)	192.3 (139.4)
Wilson *B* (Å^2^)	25.1	24.8	24.9	25.0	27.2	26.7

**Table:** ‘High’ or ‘low’ in the dataset name indicates the pump laser energy, and ‘50 µm’, ‘large’ or ‘small’ the aperture size, see Table 3. Values in parentheses are for the high resolution shell data.

	Dataset				
	SOS (50 µm) low	SOS (50 µm) high	MISP (large) high	MISP (large) low	MISP (small) high
Space group	*I*222	*I*222	*I*222	*I*222	*I*222
Unit-cell parameters (Å, °)	93.8, 105.0, 158.8, 90.0, 90.0, 90.0	93.8, 105.0, 158.8, 90.0, 90.0, 90.0	94.8, 105.4, 158.5, 90.0, 90.0, 90.0	94.8, 105.4, 158.5, 90.0, 90.0, 90.0	94.7, 105.4, 158.5, 90.0, 90.0, 90.0
No. of indexed lattices	68168	55165	46485	56455	17759
Resolution limits (Å)	10.00–1.71 (1.75–1.71)	10.00–1.71 (1.75–1.71)	10.00–1.75 (1.80–1.75)	10.00–1.75 (1.80–1.75)	10.00–1.86 (1.91–1.86)
*I*/σ(*I*)	6.8 (1.4)	6.1 (1.2)	7.8 (1.2)	8.8 (1.5)	5.8 (1.5)
*R*_split_ (%)	10.6 (85.1)	12.1 (96.5)	8.2 (93.7)	7.5 (81.3)	12.1 (74.8)
CC^1/2^	0.984 (0.524)	0.978 (0.422)	0.991 (0.404)	0.992 (0.116)	0.98 (0.594)
CC*	0.996 (0.829)	0.994 (0.771)	0.998 (0.759)	0.998 (0.457)	0.995 (0.863)
Completeness (%)	100.0 (100.0)	100.0 (100.0)	100.0 (100.0)	100.0 (100.0)	100.0 (100.0)
Multiplicity	532.3 (333.8)	421.2 (264.4)	945.6 (648.0)	1167.5 (799.5)	446.4 (324.3)
Wilson *B* (Å^2^)	26.2	26.2	29.3	28.7	28.5

**Table 2:** Data collection statistics for dark data Values in parentheses are for the high resolution shell data.

	Dataset			
	LCP (LCP) dark	HVE (HEC) dark	SOS dark	MISP dark
Space group	*I*222	*I*222	*I*222	*I*222
Unit-cell parameters (Å, °)	93.9, 105.0, 158.6, 90.0, 90.0, 90.0	94.6, 105.4, 158.5, 90.0, 90.0, 90.0	93.9, 105.0, 158.7, 90.0, 90.0, 90.0	94.8, 105.4, 158.4, 90.0, 90.0, 90.0
No. of indexed lattices	30917	55966	43502	27024
Resolution limits (Å)	10.00–1.60 (1.64–1.60)	10.00–2.08 (2.13–2.08)	10.00–1.60 (1.64–1.60)	10.00–1.75 (1.80–1.75)
*I*/σ(*I*)	5.5 (1.1)	5.2 (1.5)	6.0 (1.2)	6.5 (1.1)
*R*_split_ (%)	13.1 (99.5)	14.5 (72.2)	12.0 (94.5)	10.1 (101.0)
CC^1/2^	0.973 (0.432)	0.976 (0.566)	0.978 (0.444)	0.987 (0.361)
CC*	0.993 (0.777)	0.994 (0.850)	0.995 (0.784)	0.997 (0.728)
Completeness (%)	100.0 (100.0)	100.0 (100.0)	100.0 (100.0)	100.0 (100.0)
Multiplicity	404.0 (268.2)	208.0 (148.8)	399.0 (264.9)	647.8 (443.9)
Wilson *B* (Å^2^)	24.3	32.1	24.9	28.8

**Table 3:** Photoproduct yields (occupancies of the cleaved fatty acid) Occupancies were determined by ensemble refinement using all available data as well as 17000 light images and 27000 dark images. The indicated standard deviations were determined by bootstrapping with 100 samples each (Gorel *et al.*, 2021 ▸). This gives an estimate of the stochastic error in the determined occupancies. However, the differences between occupancies determined from all data and from 17000/27000 lattices show that there is also a systematic component to the error and that the true total errors are likely to be larger. Given the results presented here, we estimate this total error to be of the order of 5–7 percentage points. PP stands for photoproduct and occ is the occupancy determined by ensemble refinement.

Dataset	Laser fluence (mJ cm^−2^)	PP yield predicted theoretically†	PP occ (all lattices)	Percentage of theoretical maximum	No. of lattices (light/dark‡)	PP occ for 17000 light and 27000 dark lattices	Percentage of theoretical maximum
MISP (large)§	35		0.45 ± 0.02		46485 / 27024	0.44 ± 0.03	
MISP (large)	7	0.370	0.23 ± 0.02	62 ± 5	56455 / 27024	0.26 ± 0.03	70 ± 8
MISP (small)	35		0.44 ± 0.03		17759 / 27024	0.46 ± 0.04	
HVE (LCP)1	35		0.44 ± 0.03		37739 / 30917	0.39 ± 0.04	
HVE (LCP)1	7	0.353	0.28 ± 0.03	79 ± 8	38240 / 30917	0.24 ± 0.04	68 ± 11
HVE (LCP)2	35		0.40 ± 0.03		38446 / 30917	0.44 ± 0.04	
HVE (LCP)2	7	0.353	0.26 ± 0.03	74 ± 8	42115 / 30917	0.27 ± 0.04	77 ± 11
HVE (HEC)¶	35		0.34 ± 0.03		38728 / 55966	0.31 ± 0.05	
HVE (HEC)¶	7	0.346	0.22 ± 0.03	64 ± 9	38229 / 55966	0.19 ± 0.04	54 ± 12
SOS (50 µm)	98		0.38 ± 0.03		68168 / 43502	0.41 ± 0.05	
SOS (50 µm)	244		0.45 ± 0.03		55165 / 43502	0.38 ± 0.04	

† Photoexcitation probabilities were determined for 7 mJ cm^−2^ (0.5 photons per chromophore) as described in Appendix *B*[App appb]. They depend on the relative orientations of the isoalloxazine transition dipole moment, on the polarization vector of the pump laser and its intensity, and on the quantum yield, 0.8 for FAP (Sorigue *et al.*, 2021 ▸).

‡ True dark data (no laser) were used.

§ Data were collected on MISP chips with large or small apertures.

¶ In the HEC maps, a peak of unknown origin is found in close proximity to the substrate C2 atom, at the position of which map values are calculated to determine the light-state occupancy. For the HEC datasets we therefore used a position halfway between the C1 and C2 atoms for this purpose, where there are no interfering peaks.

## Data Availability

The refined dark, light and intermediate models, together with the structure factor amplitudes for all datasets listed in Table 3 ▸ and the scripts used for analysis, will be deposited at Zenodo and https://CXIDB.org within 12 months after publication of the current work.

## Appendix A Validation of the occupancy retrieval method

In order to validate our occupancy determination method, we first needed to be able to simulate realistic diffraction data. While perfect structure factor amplitudes can easily be calculated, these are unrealistic as they lack experimental errors. To model realistic errors for simulated structure factor amplitudes, we first investigated the errors of actual data sets. Structure factor amplitudes (and their standard deviations) collected from light-excited crystals were divided into 80 resolution bins and for each bin the average fractional error was calculated as 〈σ*F*/*F*〉. Plotting these values as a function of bin resolution revealed an approximately exponential fall off. This fall off was modeled with a simple function [Fig. 5 ▸(*a*)],

where *d* is the resolution and *A*, *b* and *c* are fitting parameters. The resulting error model was then used to generate errors for the calculated test data by drawing, for each reflection, a random error from a Gaussian distribution with the appropriate standard deviation as calculated using the average fractional error predicted for that reflection’s resolution by the error model. The calculated test data were then modified by these random errors. This resulted in very realistic test data, as shown by the fact that electron-density maps derived from dark and light test data produced in this way looked very similar to maps calculated from real observed data.

We then produced models of partially photolyzed FAP structures by combining a dark state and a photolyzed state PDB file, setting the occupancy of the photolyzed state to values of *x* = 0.1, 0.2, 0.3, 0.4 and 0.5, and calculating test data with realistic errors as described above. These data were then subjected to the occupancy determination method described above, starting by performing structure factor extrapolation to determine a separate light structure for each data point. For each tested occupancy this was performed three times, with different seeds for the random number generator used to generate errors. Finally, all retrieved occupancies were plotted against the actual occupancy used to calculate the test data. The resulting plot shows that the method retrieves the true occupancy faithfully, with in most cases an error of just a few percentage points [Fig. 5 ▸(*b*)]. There appears to be no dependence of this error on the occupancy itself.

We next investigated to what extent the retrieved occupancy depends on the number of images in the data. To this end, we took the MISP (large) 35 mJ cm^−2^ data and prepared partial datasets from 10000, 15000, 20000, 25000, 30000, 35000, 40000 and 45000 randomly selected images. The full data set contains 46 485 images. We then repeated the occupancy determination (including the initial extrapolation step) on each of these data sets. The resulting occupancies are shown in Fig. 6 ▸. At first glance, lower numbers of images in the light data set appear to reduce the apparent occupancy retrieved by ensemble refinement. However, given the error estimates from the simulations and from bootstrapping real data, the apparent underestimation when reducing the number of images is not statistically significant. However, comparing the error estimates obtained from the full data sets with those obtained with reduced image numbers (Fig. 4 ▸) shows that reducing the number of images results in additional variability in the occupancies obtained. Obviously, occupancy estimates obtained with larger numbers of images are likely to be closer to the true occupancies.

Perhaps unsurprisingly, the occupancies found by ensemble refinement depend strongly on the results of the extrapolation step, which determines the light-state part of the ensemble used for occupancy determination. We have, for each occupancy determination reported here, including those performed on individually bootstrap-resampled datasets, performed a dedicated structure factor extrapolation and subsequent light-state refinement.

The errors introduced during the extrapolation step are considerable at low occupancies. During the validation calculations, we found that some jobs attempting to retrieve a preset occupancy of 0.1 crashed, as the light-state structure extracted during the extrapolation step was so poorly determined that ensemble refinement appeared to result in negative occupancies, bringing the process to a halt. In the end, we had to run five jobs from which only three finished. It is important to note that this happened with simulated data, of which the errors mirrored those from very good data (the 1.71 Å resolution SOS 110 nJ data, with 55000 images in the light data set and 43000 images in the dark data set). In this study, the occupancies of the experimental data sets are much higher than 0.1 (the threshold level for detection), but this analysis does illustrate the difficulty (if not unfeasibility) of determining high-quality light-state structures from low-occupancy data using extrapolation. In such cases, one would be tempted to pursue higher occupancies by increasing the pump laser fluence. However, as was discussed before (Grünbein *et al.*, 2020 ▸; Barends *et al.*, 2024 ▸), this could lead to multiphoton artifacts. The only way forward in such cases would be, therefore, to increase the data signal-to-noise ratio by collecting more images (Gorel *et al.*, 2021 ▸).

## Appendix B Calculation of expected photoproduct yields

In order to predict photoproduct yields in a pump–probe serial crystallography experiment, various factors need to be considered. First, one needs to account for the orientation of the transition dipole moments of the chromophores and how they relate to the electric field vector of the pump light. In the current case, the chromophore is FAD and the direction of the FAD transition dipole is in the plane of the flavin ring system, parallel to its long axis [Fig. 7 ▸(*a*)]. This allows for the determination of the direction of this dipole in the unit cell from the protein crystal structure and, by applying the space group’s symmetry operators, the directions of the transition dipoles in symmetry-related molecules can be determined as well. By applying the orientation matrices of all crystals in a dataset, the distribution of transition dipole orientations with respect to the laboratory frame can be determined. For efficient excitation by circularly polarized light in the *XY* plane, as in the experiments described in this paper, the transition dipoles should be parallel to that plane (*i.e.* perpendicular to the pump beam); orientations perpendicular to the *XY* plane (parallel to the pump beam) will have no excitation. The probability of a FAD molecule being excited by circularly polarized light in the *XY* plane is proportional to sin^2^α, where α is the angle between the transition dipole and the *Z* axis (along the pump and X-ray beam) (Kliger *et al.*, 1990 ▸).

The distribution of orientations of the transition dipole vectors can be visualized by displaying the density of the normalized vectors’ end points on a unit sphere. For the HEC data [HVE injection, Fig. 7 ▸(*b*)], some preferential orientation almost parallel to the beam direction can be observed and the average value of sin^2^α can be calculated to be 〈sin^2^α〉 = 0.61 (for reference, for a perfectly isotropic distribution of orientations this yields 0.66666…). An essentially identical distribution is observed for LCP [also HVE injection, Fig. 7 ▸(*c*)], with a value for 〈sin^2^α〉 of 0.63. For the MISP chip data [Fig. 7 ▸(*d*)], 〈sin^2^α〉 is marginally higher at 0.68. However, for the SOS chip data [Fig. 7 ▸(*e*)], a clear preferential orientation of the transition dipole moments almost parallel to the beam is seen, which reduces the excitation efficiency. Indeed, calculating 〈sin^2^α〉 shows it to be 0.54, lower than for the other two sample presentation methods. It should be noted, however, that this number is likely to be somewhat inaccurate, as the precise angle between the pump laser beam and the X-ray beam during the experiment is not exactly known. Nevertheless, this analysis demonstrates how preferential orientation of crystals can affect the excitation probability.

Having calculated the value of 〈sin^2^α〉, one needs to account for the propagation of the pump light through the crystal and the fall off in its intensity with penetration depth. In order to do so, it is useful to consider the following pair of coupled equations:

Here *x* is the depth inside the crystal, *t* is time, *I* is the pump power density (in W cm^−2^),  is the pump photon energy (in J), *N*_0_(*x*, *t*) is the concentration of molecules in the unconverted ground state (in number of molecules per cm^3^), Φ is the quantum yield (0.8 for FAP) and σ_eff_ is the effective inter­action cross section. This last quantity can be derived from the molar absorption coefficient ɛ for FAD and sin^2^α,

Here σ is the isotropic absorption cross section (cm^2^) and ɛ is the molar absorption coefficient (*M*^−1^ cm^−1^) of a FAD molecule. Both quantities assume random orientation of the FAD transition dipole moment, like in the solution phase. *N*_A_ is Avogadro’s constant. As defined here, the σ_eff_ term takes into account that the pump light is circularly polarized. It has to be noted that the isotropic molar absorption coefficient (ɛ = 11300 *M*^−1^ cm^−1^ for FAD) assumes random orientations of the absorbing molecules (Lakowicz, 2006 ▸). These considerations stem directly from the product between the electric field and the transition dipole moment in the considered geometry.

Equations (1) and (2) are a variation of the equations used to describe pulse propagation in a lasing medium, solved by Frantz & Nodvik (1963 ▸). Since our work concerns nanosecond pulses, no stimulated emission is expected from the FAD molecule. Therefore, we will consider only the transition to the excited state and assume that a molecule that is excited in a nonproductive way (decays instead of initiating the de­carboxyl­ation) immediately returns to the population of non-excited molecules. For the sake of simplicity, we also assume that all the molecules absorb light in the same way, even if they are in the active population, whose concentration we define as *N*_1_(*x*, *t*). Note that these equations are already in the frame of reference of the pump pulse, which greatly simplifies the photon transport equation [see Frantz & Nodvik (1963 ▸) for more details].

If we replace *N*_0_(*x*, *t*) with *N*_0_(*x*, 0) in equation (2), we can solve it independently of equation (1). The *N*_0_(*x*, 0) term is equal to the initial concentration of the chromophores in the crystal [*N* = *N*_0_(*x*, *t*) + *N*_1_(*x*, *t*)] so *N*_0_(*x*, *t* = 0) = *N*. This is the approximation that all the molecules absorb light in the same way mentioned above, regardless of whether they are in the *N*_0_ or *N*_1_ population. Equation (2) becomes

By integration over *x*, this results in the classical Beer–Lambert term,

*I*(0, *t*) is the power density profile of the incident pump. Applying this to equation (1) results in

By integration over time, we obtain

Note that the remaining integral is just the fraction of the pulse energy density (J cm^−2^) up to a certain moment in time. Let us define this as

 is the total pulse energy density (J cm^−2^). This leads to the following expression for the ground-state population,

Finally, one can calculate the occupancy at a position *x* within the crystal and a moment *t* during irradiation,

This formula can be used to determine the occupancies of all slices of the crystal at any given time during irradiation. By setting  and averaging over *x*, one can obtain the expected occupancy of the light state in the crystal which is characterized by the FAD transition dipole orientation described by sin^2^α. By substituting sin^2^α = 〈sin^2^α〉 in equation (3) and using the values for 〈sin^2^α〉 obtained from the crystal orientation matrices, one obtains values of σ_eff_ that are corrected for the transition dipole moment orientations. Using these values in equation (10), one can obtain the occupancy for a ‘representative’ crystal for the transition dipole moment distribution determined in the experiment, assuming that no pump light is scattered before hitting the sample.

The results of an example calculation are shown in Fig. 8 ▸. For our low-fluence datasets (7 mJ cm^−2^ pump energy density), assuming 20 µm crystals, ɛ = 11300 *M*^−1^cm^−1^, Φ = 0.8 and a protein concentration *c* = 0.0091 *M*, one obtains expected occupancies without scattering losses of 0.370, 0.353 and 0.346 for MISP, LCP and HEC, respectively (Table 3 ▸).
