# Supplementary material for: Quantifying optical scattering losses in pump–probe serial femtosecond crystallography experiments
Source: IUCrJ. 2026 Jul 16;13(Pt 5):606–22. doi: 10.1107/S2052252526005403 (PMC13548834; doi:10.1107/S2052252526005403)
Supplement: Supplementary file 1 [file m-13-00606-sup1.pdf]

IUCrJ

Volume 13 (2026)

Supporting information for article:

Quantifying optical scattering losses in pump-probe serial femtosecond crystallography experiments

Thomas R.M. Barends, Alexander Gorel, Stanisław Niziński, Martin V. Appleby, Marco Kloos, Soshichiro Nagano, Mario Hilpert, Florian Dworkowski, Claudio Cirelli, Emma V. Beale, Elisabeth Hartmann, Camila Bacellar, Robert L. Shoeman, R. Bruce Doak, John H. Beale, Philip J.M. Johnson and Ilme Schlichting

S1. Supplementary Text: Practical considerations for pump-probe experiments

The goal of pump-probe experiments is to characterize the properties of the light-triggered species occurring during the reaction investigated (we are excluding investigations using caged compounds since they focus on the reactions occurring with or enabled by the uncaged compound). The pump laser energy density should be chosen such that the excitation occurs in the linear photoexcitation regime to ensure study of the biologically relevant photoreaction and/or to allow meaningful comparison with spectroscopic investigations. To facilitate interpretation of the light-induced signal, it is often helpful to optimize the fraction of the light-triggered species. Due to the typically very high chromophore concentration in macromolecular crystals (10-50 mM) this is particularly problematic in crystallographic studies. It is essential to use crystals whose dimensions are smaller than the 1/e penetration depth of the photoexciting light, otherwise a large fraction of the crystals does not "see" any light and the F_{obs}^{light} - F_{obs}^{dark} differences are too small to analyze [see Figure Extended Data Figure 2 in (Barends *et al.*, 2024)]. Shifting of the excitation wavelength away from the ground state absorption peak to allow usage of thicker crystals should be done only if it has been shown spectroscopically that this neither affects the photoproduct yield nor introduces nonlinear effects (see for example (Hutchison *et al.*, 2016)). Thus, one of the most important variables for a successful pump probe experiment is to use suitably-sized crystals with a narrow size distribution (Grünbein *et al.*, 2020). A suitable laser energy/(power) density for excitation in the linear regime can be estimated from spectroscopic investigations establishing the linear regime [typically ~0.3 photons/chromophore see for example Extended Data Figure 1 in (Barends *et al.*, 2024) or Figure 2(*d*) in (Nass Kovacs *et al.*, 2019)] and the known chromophore concentration in the crystals, the molar extinction coefficient of the chromophore and the laser parameters (focal size, laser energy, wavelength, laser pulse duration). When performing SFX-based laser power titrations it is important to include several energy densities that correspond to the linear regime (*i.e.*, << 1photon/chromophore). This can be very difficult if not impossible for crystals that are thicker than the 1/e light penetration depth. However, in the case of thick crystals, higher light intensity penetrates deeper into the crystal, apparently increasing the photoproduct yield while also increasing multiphoton absorption at the "front" of the crystal facing the pump light. It is possible thus that a linear dependence of the apparent photoproduct yield and laser energy density is observed, but this could be the one of the multiphoton photoproducts [see Figure 1(*b*), Extended Data Figure 1(*d*), in (Barends *et al.*, 2024)]. Vigilance is thus required.

A hotly debated issue is the fraction of the pump light lost due to scattering effects. Published estimates range from 99 % (Carrillo *et al.*, 2021) to ~20 % (Grünbein *et al.*, 2020, Nass Kovacs *et al.*, 2019). The latter estimates are in line with recently published all optical investigations (Nizinski *et al.*, 2025) and the results reported here. However, in all investigations reporting "low" scattering

losses great care was taken to avoid situations that inherently result in light scattering. A prominent counter example is Super Lube™ (and related materials), a convenient and cheap embedding material (Sugahara *et al.*, 2015, Sugahara *et al.*, 2020). The hydrophobic character of grease-like embedding materials results in a phase separation of the aqueous liquid surrounding the crystals and the encasing grease, resulting in a plethora of strongly scattering water droplets with sizes smaller than or comparable to the visible light wavelength [see Figures S20- S22 in (Nizinski *et al.*, 2025)]. Since this effect strongly depend on water content – determined by the amount of liquid surrounding the embedded crystals - it is also difficult to reproduce. This feature is in line with the observation of significant light contamination, see for example (Li *et al.*, 2021). As evidenced by Monte-Carlo light scattering simulation (Nizinski *et al.*, 2025), very high scattering will result in a highly nonuniform light intensity profile along the light direction in the sample (although the average light intensity is not expected to decrease dramatically). Embedding materials like Super Lube™ should therefore be avoided, as their use results in ill-defined photoexcitation conditions. We recommend to use only optically transparent embedding media such as HEC, LCP or hyaluronic acid (Sugahara *et al.*, 2016).

Various approaches have been tried to estimate the losses due to scattering, unfortunately this is a complex endeavor and it requires specialized equipment (Nizinski *et al.*, 2025). Therefore, we recommend a simple qualitative test on the chosen embedding medium (ideally but not necessarily with crystals) before the beamtime (after the syringe-mixing and other procedures). To this end, a small amount of the embedding medium is placed between two flat transparent windows, so that one can look through the sample layer (the sample should have at least ~1cm^2 area, see Figure S6). The sample layer thickness should be similar to one that will be used in the planned experiment (HVE jet diameter, in case of SOS data collection one can use the SOS chip as it is, provided they are available before the beam time). The thickness can be adjusted by spacers cut from PTFE sheets of proper thickness or by layers of TESA™ film tape (one layer corresponds to ~ 50-60 µm, but it is recommended to check the manufacturer webpage or measure it with calipers). Alternatively, one can use demountable optical cells with defined thickness (such as the ones from Hellma). If one can clearly see objects and their edges located behind the filled glass slide through this layer, it means that a large fraction of the light travels through without scattering and the expected light losses are certainly much lower than 30% [see Figure S21 (a,c) in (Nizinski *et al.*, 2025)]. In case one cannot see clearly through the sample layer [see Figures S20(b-d) in (Nizinski *et al.*, 2025)], scattering is significant and may result in high inhomogeneity of the light intensity along the propagation direction in the sample, as well as light contamination of not-directly exposed sample regions. This simple test can be used to optimize the sample preparation before the beamtime and to do a fast check directly before the experiment. Additionally, one should investigate the test sample layer under a light microscope and check for “features” in the transparent sample layer, such as metal debris [see Figure S10 (Nizinski *et al.*, 2025)].

The next step is to embed microcrystals into the chosen optically transparent viscous matrix, dispense some of the mixture between glass slides and check under a microscope whether the crystals look similar to before they were embedded (see Figure S1). This check should be repeated after at least the time required to collect diffraction data of a HVE syringe filling or a chip. If no differences are observed, the next step would be to test the diffraction properties of the crystals before and after embedding.

The resolution of the diffraction data should not drop significantly upon crystal embedding, unit cell distributions should be narrow and, ideally, not differ from that of the native crystals. In case of unit cell changes (in particular with embedding time) a different embedding medium should be chosen. In case of a phase transition of the crystals upon embedding, it needs to be investigated whether this affects the crystal or structural properties to an extent that affect the interpretation of the experiment. The HEC embedding of FAP crystals is an example of a very poor match between crystals and embedding medium, the crystals are clearly on the verge of disappearing; we included these observations in the manuscript as an example where things did not work out [Figure S1(*a*-*c*) vs Figure S1(*d*), Table S1,2].

If all of these precautions are taken and if the crystals have the right size (distribution) for efficient photoexcitation (*i.e.*, they are small enough), we recommend to routinely assume ~20-30% scattering/reflection losses when deciding on a pump laser energy. In general, we also do not consider SFX pump laser excitation energy titrations to be necessary. Power titration experiments performed by spectroscopic approaches combined with calculation of number of absorbed photons per molecule (tools presented in (Grünbein *et al.*, 2020) are sufficient.

Last but not least, an important decision concerns the sample injection approach. This does not only have implications on the amount of material needed (Martiel *et al.*, 2019, Grünbein & Kovacs, 2019) but also on preferred crystal orientations. Rod-like crystals flow align in liquid jets and lie flat on chip surfaces, as do plate-like crystals as also observed in the current study (see also (Zarrine-Afsar *et al.*, 2012). These preferred orientations do not only affect completeness of the diffraction data (sampling of reciprocal space) but can also have profound effects on photoexcitation yields as described in the Appendix B.

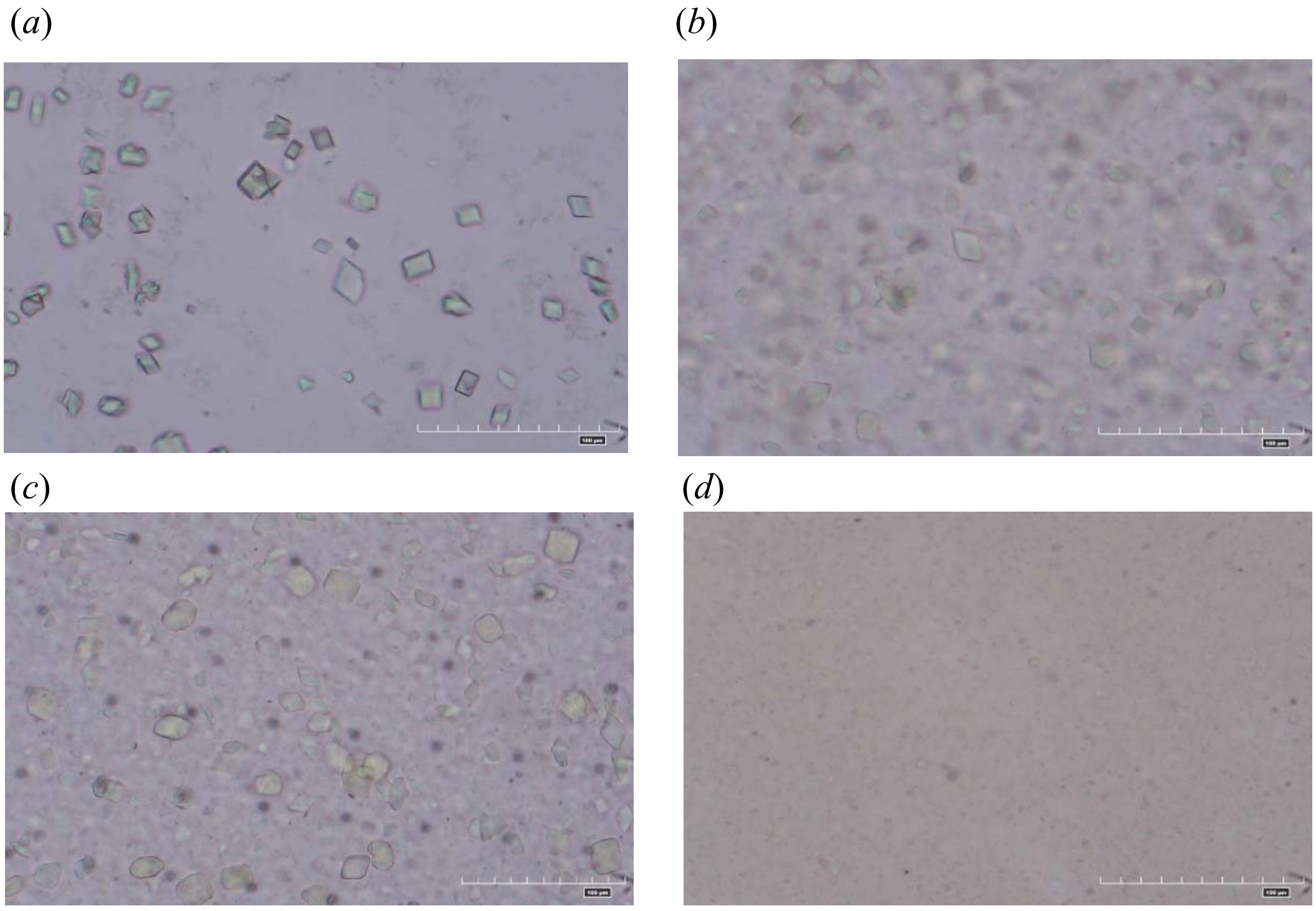

Figure S1 **Micrographs of FAP microcrystals**. (*a*) Before any manipulation in mother liquor (50 mM NaCitrate pH 5.5, ~ 20% PEG 3,350). The crystals have sharp, well-defined edges. (*b*) FAP microcrystals embedded in LCP before loading into the HVE injector. FAP crystals are mechanically sensitive, upon mixing into LCP the edges round off. (*c*) FAP microcrystals embedded in LCP, mounted in an SOS chip, following SFX data collection using 50 × 50 µm X-ray step sizes within and between scan lines. FAP crystals are mechanically sensitive, upon mixing into LCP the edges round off. Note the crystal alignment with preferentially one face perpendicular to the viewing axis. (*d*) FAP microcrystals embedded in HEC. 14% HEC is rather stiff, the mechanically rather sensitive FAP crystals fractured upon mixing. This is not caused by the mixture itself since a few percent of HEC are well tolerated; the crystals keep their sharp edges and do not fracture. In all cases, the length of the white scale bar is 100 µm with 10 µm subdivisions.

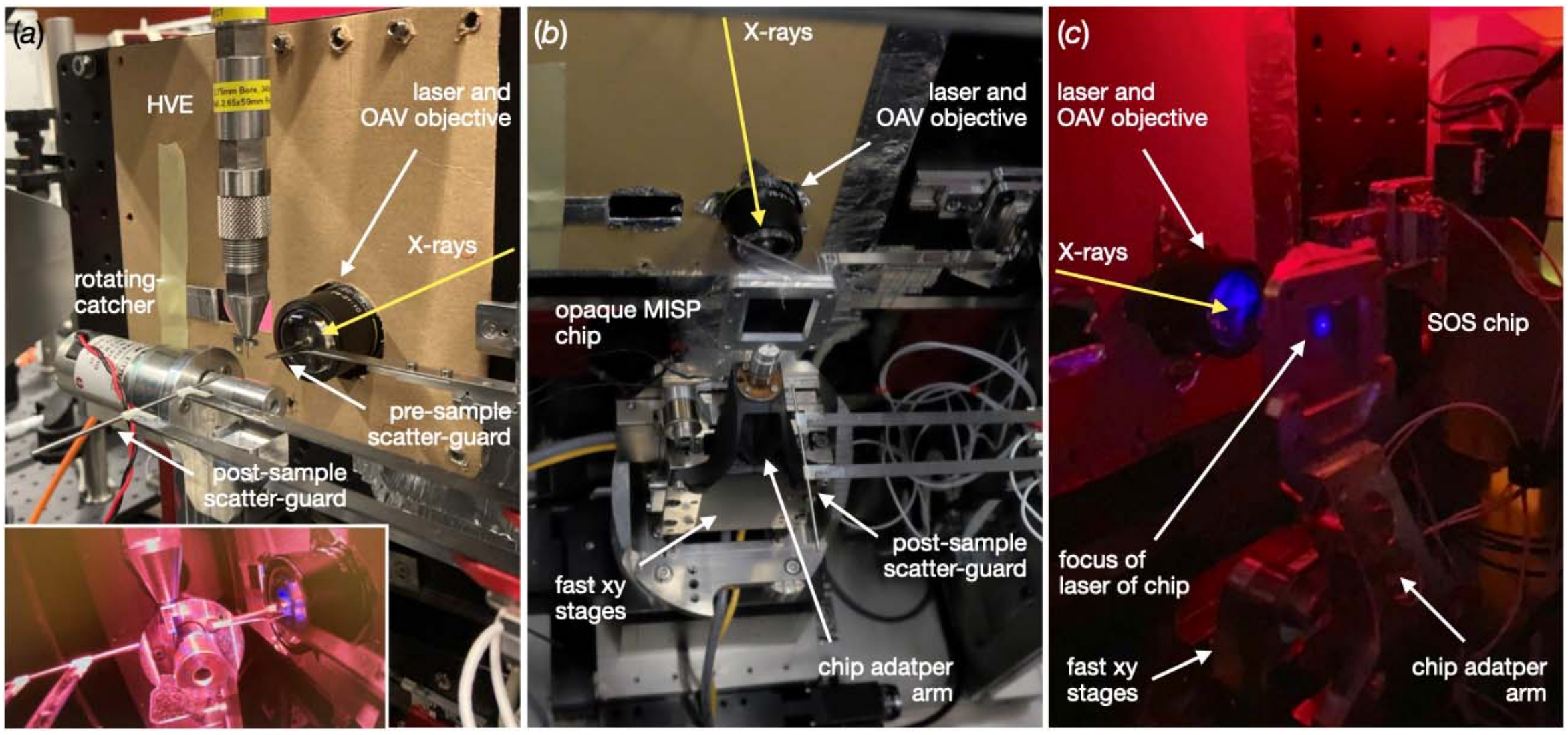

Figure S2 **The sample delivery systems used in the experiment.** (*a*) The MPI HVE injector and rotating catcher (Doak *et al.*, 2023) mounted at the sample position with both the pre- and post-sample scatter-guards in 'data collection' position. The bottom inset is an orthogonal view looking along the rotating-axis of the catcher during a measurement. The 470 nm laser-light can be observed coming from the objective lens and hitting the extruded jet. (*b*) The opaque MISP chips in their holders at the sample position. The pre- and post-sample scatter-guards are in a 'sample alignment' position. (*c*) An SOS chip at the sample position, with the 470 nm laser illuminating part of the chip. The chip adapter arm was changed to accommodate the different chips, otherwise the device was the same as for the MISP chips.

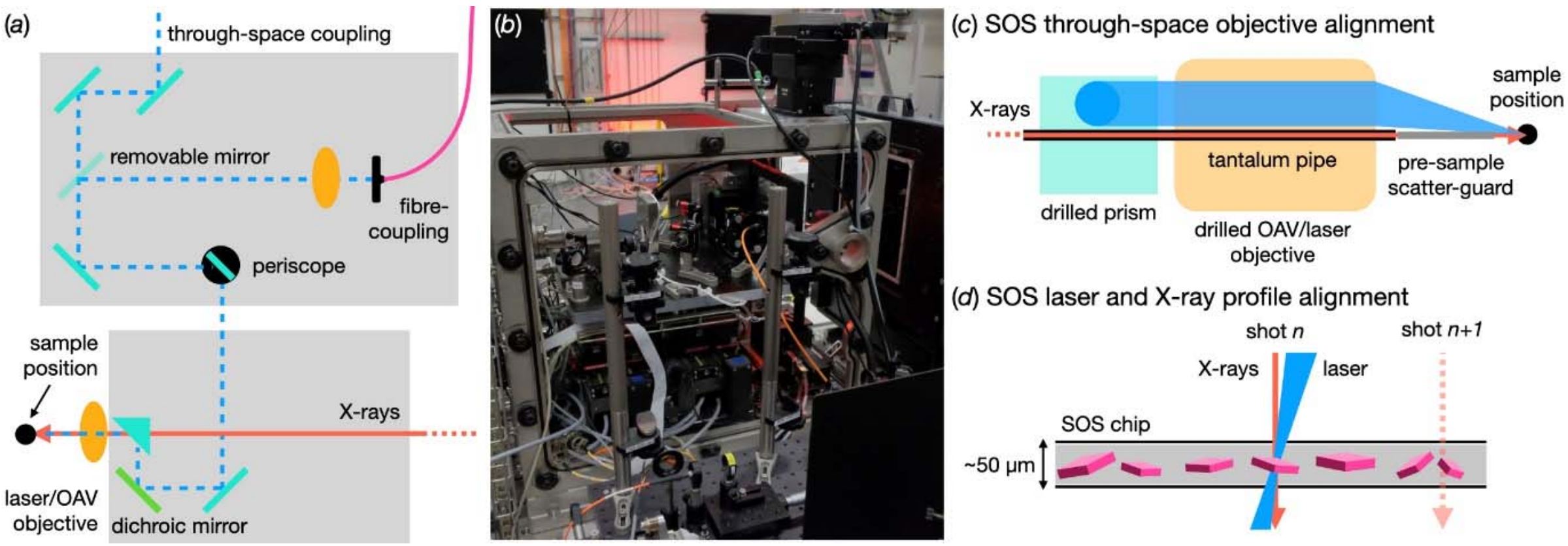

Figure S3 **An overview of the different laser couplings and the specific challenges associated with the SOS chips.** (*a*) A schematic of the fibre and through-space laser couplings to the sample position. (*b*) An image of coupling with the HVE mounted at the sample position. (*c*) Alignment of the through-space laser through the drilled objective such that the centre of the Gaussian was preserved. (*d*) A schematic of the X-ray and pump laser overlap in the SOS chip. Due to their comparable size, the Gaussian profiles of the lasers must overlap within a few micrometres to ensure correct excitation.

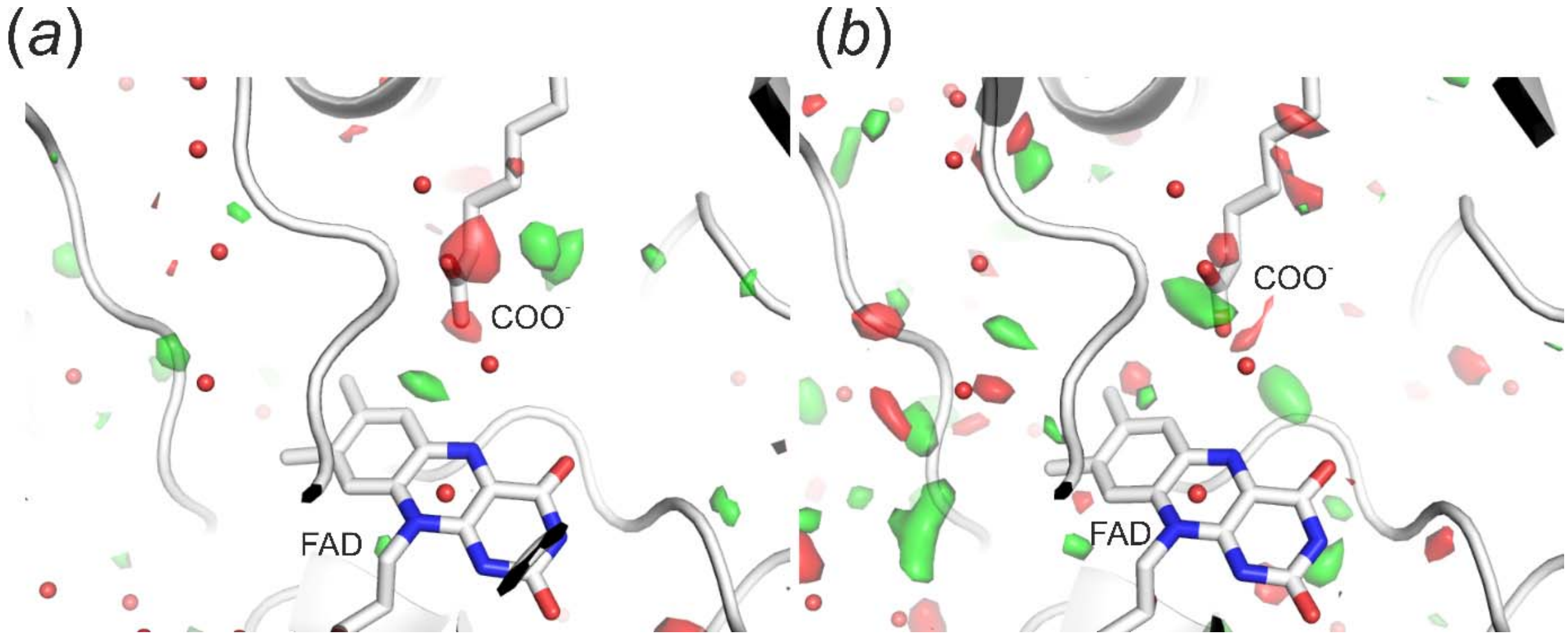

Figure S4 (a) Sensitivity limit of the F(interleaved dark)-F(totally dark) difference maps due to the low number of interleaved dark images. Shown is the q-weighted difference map contoured at +3.0σ (green) and -3.0σ (red) calculated from synthetic data sets with the same numbers of images as in the actual experiments, as described in the text. At 10% occupancy of the photo-dissociated state, the expected negative density peaks on the substrate's carboxylate group (COO^-) are the more prominent peaks in the active site. (b) q-weighted F(interleaved dark)-F(totally dark) difference map of the 50 μm SOS data set contoured at +3.0σ (green) and -3.0σ (red). No interpretable features are visible. The substrate and the FAD are shown as sticks.

a) *b)*

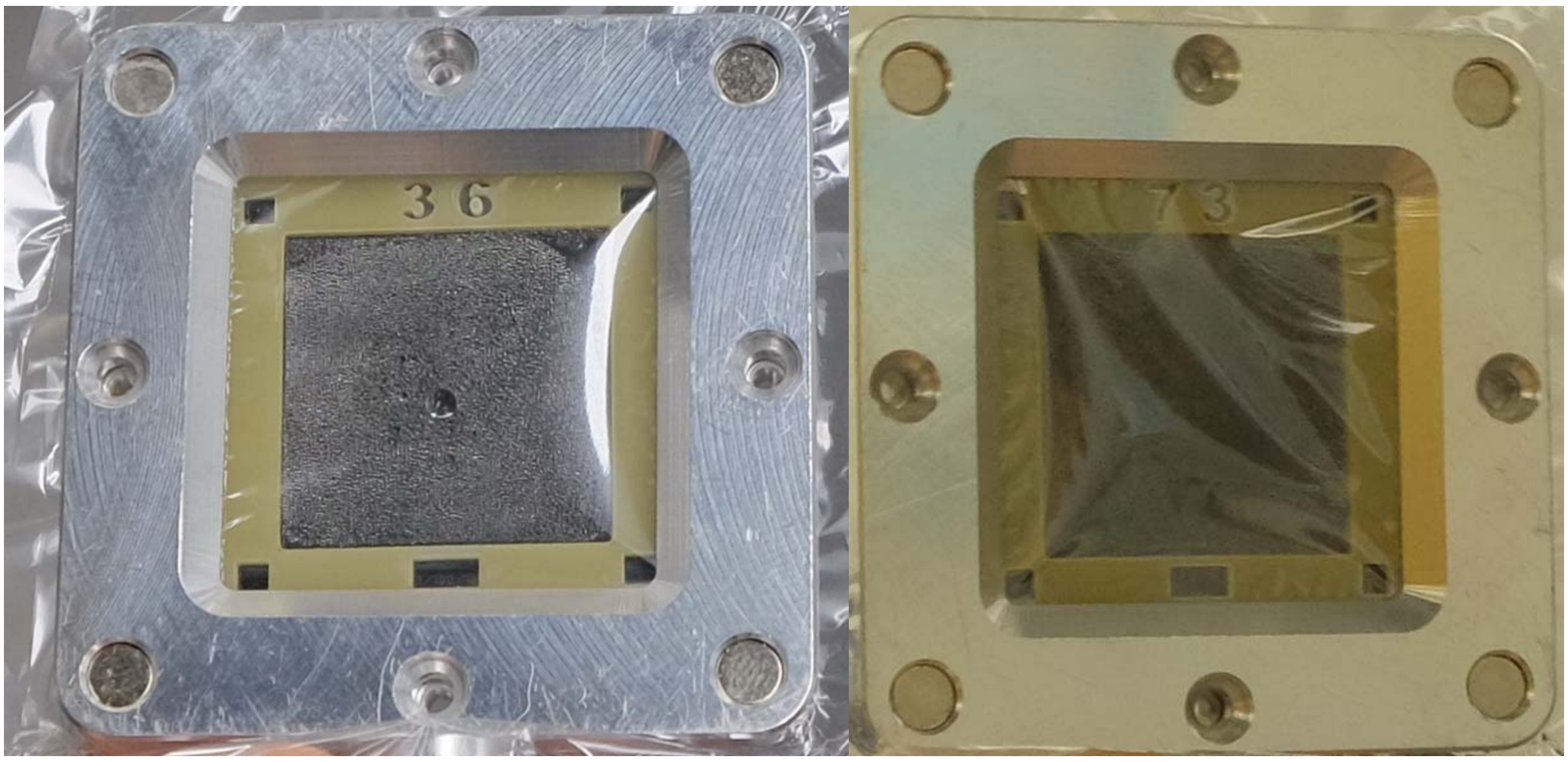

Figure S5 (a) A loaded MISP chip showing many droplets of condensation on the Mylar foil facing the pump laser. **(b)** MISP chips have no built-in mechanism for stretching the Mylar films protecting against dehydration.

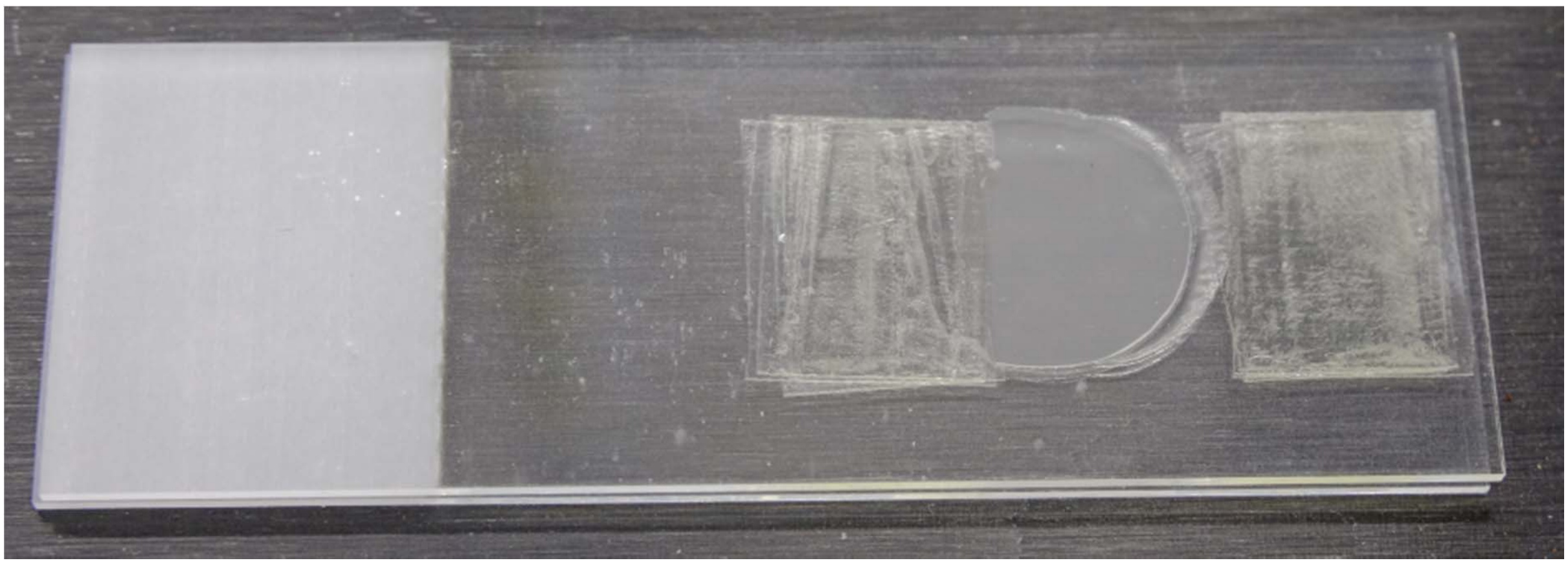

Figure S6 Example preparation of a Super Lube™ PTFE sample mounted between two microscope glass slides. The sample thickness is 280 µm; the spacers consist of 5 layers of Tesa™ Film crystal clear, # 57766. Even without mixing with a crystal slurry, the Super Lube™ PTFE sample layer is completely opaque, indicative of strong scattering.
